# Three-stage processing of category and variation information by entangled interactive mechanisms of peri-occipital and peri-frontal cortices

**DOI:** 10.1038/s41598-018-30601-8

**Published:** 2018-08-15

**Authors:** Hamid Karimi-Rouzbahani

**Affiliations:** 1 Department of Electrical Engineering, Shahid Rajaee Teacher Training University, Tehran, Iran; 2 Perception in Action Research Centre & Department of Cognitive Science, Faculty of Human Sciences, Macquarie University, Sydney, NSW 2109 Australia; 3 ARC Centre of Excellence in Cognition and Its Disorders, Macquarie University, Sydney, NSW 2109 Australia

## Abstract

Object recognition has been a central question in human vision research. The general consensus is that the ventral and dorsal visual streams are the major processing pathways undertaking objects’ category and variation processing. This overlooks mounting evidence supporting the role of peri-frontal areas in category processing. Yet, many aspects of visual processing in peri-frontal areas have remained unattended including whether these areas play role only during active recognition and whether they interact with lower visual areas or process information independently. To address these questions, subjects were presented with a set of variation-controlled object images while their EEG were recorded. Considerable amounts of category and variation information were decodable from occipital, parietal, temporal and prefrontal electrodes. Using information-selectivity indices, phase and Granger causality analyses, three processing stages were identified showing distinct directions of information transaction between peri-frontal and peri-occipital areas suggesting their parallel yet interactive role in visual processing. A brain-plausible model supported the possibility of interactive mechanisms in peri-occipital and peri-frontal areas. These findings, while promoting the role of prefrontal areas in object recognition, extend their contributions from active recognition, in which peri-frontal to peri-occipital pathways are activated by higher cognitive processes, to the general sensory-driven object and variation processing.

## Introduction

Humans can recognize the categories of objects in fractions of a second with remarkable accuracy^1,2^. This ability seems more outstanding considering that an individual object can produce almost an infinite number of distinct images on the retina imposed by the variations that it undergoes (e.g. size, position, pose, etc.) as well as the variations in the surrounding environment^3^ (e.g. background, lighting direction, etc.). This has motivated many researchers to investigate the neural underpinnings of invariant object recognition; a sensory-cognitive brain process which is continuously employed in everyday life.

The general consensus is that, the main processing infrastructures of the brain which underlie object category processing are the ventral and the dorsal visual streams^4–6^. The ventral stream starts from V1 and ends up at anterior inferior temporal cortex (IT)^7,8^ and the dorsal stream starts from V1 and continues to parietal and areas in middle temporal cortices^9–11^. In object recognition, linearly-separable representations of objects as well as other accompanying aspects of information, which are processed by the layers of the two visual streams, are then sent to the prefrontal cortex for final classification of representations into distinct categories^12^ (e.g. categories of objects, movement directions, etc.). However, this view has been challenged by recent studies which observed category-related information in frontal brain areas, even earlier than they generally appeared in occipital and temporal brain areas after stimulus presentation^13–15^. These latter studies were triggered by a pioneering investigation which reported the encoding of categorical information in orbitofrontal cortex (OFC)^16^. However, systematic investigations are still needed to provide deeper insights into the contribution of frontal brain areas in the processing of object categories and variations as well as possible interactions between anterior and posterior brain areas.

Based on the model proposed by Bar *et al*.^14^, the orbitofrontal cortex receives low-frequency category-related information from early visual areas (e.g. V1 and V2) through magnocellular pathways^9,17^, and sends initial guesses about the possible category of the target object to higher visual areas in inferior temporal cortex (e.g. fusiform gyrus) for more rapid and accurate categorization^13–15,18^. In the study performed by Bar *et al*.^14^, the phase-locking of category-related responses was measured across occipital and orbitofrontal cortices to evaluate their functional connectivity. It was suggested that the flow of information was from occipital to orbitofrontal cortex in the window roughly from 80 to 180 ms post-stimulus and from orbitofrontal to IT cortex at later time windows (after 130 ms). However, as the mentioned study considered the average responses as representatives of the amount of information, and did not explicitly measure the flow of category information between the mentioned areas, it has remained unknown whether it was actually the ‘category’ information which was transferred between those areas or the observed phase-locking of responses represented other aspects of the neural information.

A recent study applied multivariate pattern analysis along with Granger causality analysis on magneto encephalographic (MEG) data and investigated the encoding and transfer of category information across peri-frontal and peri-occipital areas^19^. That study reappraised the model proposed by Bar *et al*.^14^, with low- and high-spatial resolution image sets presented to subjects in a recognition experiment. Nonetheless, the spatiotemporal dynamics of category encoding was drastically different from those reported in Bar *et al*.^14^: the results showed the domination of feed-forward information flow from peri-occipital to peri-frontal areas in early processing time windows (from 0 to around 500 ms post-stimulus) and the domination of feedback flows in the following time windows (from 500 ms to 1200 ms post-stimulus). Authors explained that the observed discrepancy from previous results (Bar *et al*.^14^) could have been explained by long stimulus presentation time (i.e. 500 ms) in their study which caused the domination of feed-forward information flow in early time windows^19^. Therefore, new paradigms, such as the one employed in the current study, which provides a shorter presentation time, are needed to reappraise the spatiotemporal transfer of category information between peri-occipital and peri-frontal brain areas.

Importantly, when investigating the processing of category information in the brain, one needs to always take into account the impact of object-oriented and ambient variations (e.g. size, position, pose and lighting) on categorical information and possible spatiotemporal interactions between category and variation information in the brain. The processing of these variations can facilitate human navigation and human-object interactions by providing information about the location of the object in the space (i.e. position), its viewpoint (i.e. pose), distance (i.e. size), etc. Previous studies have shown that, in almost every single processing stage of the ventral and dorsal visual pathways, the information about categories and variations are observable concurrently^20–28^. More specifically, along the ventral visual stream, the entangled V1-level categorical information becomes untangled by transformations which are implemented by the neural structures from V1 to IT^12^. On the other hand, it has been recently shown that the same neural structures untangle different conditions of individual category-orthogonal variations (e.g. size, position, pose), hence increasing ‘variation information’ along the stream^23^. In addition, the processing of some specific variations has been suggested to be one of the main reasons for the activation of feedback mechanisms in the brain^8,29–32^. However, it has remained to be known whether the frontal brain areas also participate in the processing of variation information (called ‘variation processing’ here). While a few neuroimaging studies have addressed the processing of affine variations in the brain^23,28^, the role of frontal brain areas and their possible interaction with visual areas in variation processing have remained largely overlooked by neuroimaging investigations.

In summary, this study pursues two major goals: first, to evaluate the feasibility of extracting variation information from EEG activities and to compare its temporal dynamics with that of the well-studied category-related information. Second, to investigate the spatial dynamics of variation/category processing on the brain with focus on the interaction between frontal and occipital areas in information processing.

To address these questions, I developed a whole-brain electroencephalography (EEG) recording experiment in which humans were presented with a set of visual objects which underwent levels of controlled variations. The stimuli were presented very briefly and the paradigm was designed in passive format to allow the appearance of feedback signals in early time windows^19^ and to avoid influences from higher top-down cognitive signals which usually appear during active recognition^33–35^, respectively. Using multivariate pattern analysis (MVPA), I analyzed the spatiotemporal dynamics of category and variation processing in the brain and found that the occipitotemporal, parietal and prefrontal areas were the major areas involved in the processing of categories and variations by entangled mechanisms. I also implemented a recently-proposed version of Granger causality^19^ to investigate the dynamical transactions of category and variation information between the peri-occipital and peri-frontal brain areas. Interestingly, I found three distinct stages of information transactions between these areas which supported object recognition. These results provide evidence that a set of mainly sensory-driven (task-independent) parallel interactive mechanisms across peri-occipital and peri-frontal areas process a combination of category and variation information in distinct stages of processing.

## Methods

### The dataset

The EEG dataset used here was previously utilized to investigate the role of three major signal parameters in the representation of categorical information in the brain^36^. Using a computational modelling methodology, that study showed that the average activity of EEG signals contributed most dominantly to the representation of object categories as compared to independent and dependent variability of the signals^36^. Here, I analyzed the data for its main purpose: to study the spatiotemporal dynamics of category-orthogonal processing in the brain and the contribution of frontal brain areas in that processing.

### Stimulus set

**Figure 1:**
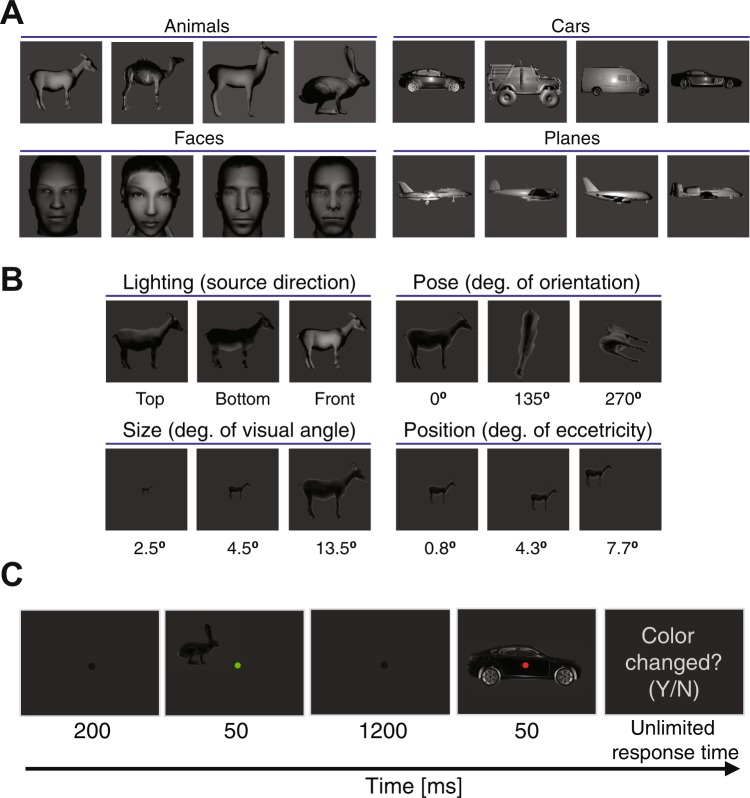
Image set and experimental paradigm. (**A** and **B**) show the object exemplars within each category (**A**) and conditions of each variation (**B**). The 3D models used to generate these images were available under a personal and commercial license (http://www.cadnav.com/help/copyright.html) and were freely downloaded from (http://www.cadnav.com). Images were processed (zoomed in and cropped) for better illustration. Extra information regarding condition are provided below it. (**C**) EEG recording paradigm with numbers indicating the presentation time of each event.

In order to investigate the processing of object categories and variations in the brain, a four-category object image set was generated with levels of controlled variations. The 3D object mesh models, used in the generation of the image set, were freely downloaded from the internet (http://www.cadnav.com) and rendered using Python commands in the freely available rendering software ‘Blender’ (https://www.blender.com). The image set consisted of four ordinary categories of objects including animals, cars, faces and planes, each of which underwent levels of variations in size, position, pose (in-depth rotation) and lighting (Fig. 1A). Each category consisted of four unique exemplars to enhance the generalizability of the image set. In order to cover natural variations of objects, which humans observe in everyday object recognition, I generated images in which objects underwent three levels of variation in size (i.e. 2.5, 4.5 and 13.5 degrees of visual angle), and positioned the objects on three different locations (i.e. with foveal eccentricities of about 0.8, 4.3 and 7.7 degrees of visual angle). I also applied three levels of in-depth orientations on the objects (i.e. 0, 135 and 270 degrees of orientation simultaneously around X, Y and Z Cartesian axes) and illuminated them from three different directions (i.e. top, bottom and front; Fig. 1B). I used a uniform lighting source for all variations except for the lighting conditions. The lighting conditions were selected in a way to present the objects in their most hard-to-recognize everyday conditions, so as to activate all primary and secondary brain mechanisms which are considered to play role in object processing (i.e. including mechanisms of peri-occipital as well as peri-frontal cortices). The final image set consisted of 192 unique images with an area of 512 by 512 pixels. In order to avoid trivial decoding results, the image set was normalized for equal across-category and across-variation average luminance and contrast. Note that the presented images in Fig. 1 are zoomed and chosen from the frontal lighting condition (i.e. 3^rd^ lighting condition) for improved visualization.

### Experimental design

I recorded the brain activities using electroencephalography as this imaging modality could provide the activities with high temporal and moderate spatial resolution. The former characteristic could pave the way for studying the highly time-dependent dynamics of object representation in the brain. I designed a passive recording paradigm in which subjects were not supposed to categorize the presented objects, but rather had to attend to the color of the category-irrelevant fixation points during the experiment (Fig. 1C). Specifically, at the beginning of each trial, a black fixation spot was presented on the center of the screen for 200 ms after which the first stimulus was presented for 50 ms. Upon the disappearance of the stimulus, an inter-stimulus interval was maintained for 1200 ms before the second stimulus was presented to the subject for 50 ms. The fixation spot remained on the center of the screen throughout the trial, but randomly switched color to either red or green across each stimulus presentation. A post-hoc verification step showed no relationship between the color of the fixation spots and the conditions used in the representational analysis. Therefore, the observed spatiotemporal dynamics of category and variation processing could not be attributed to the processing of colors in the brain.

### Subjects’ task

Subjects’ task was to decide whether the color of the fixation spot was the same or different from the first stimulus to the second (i.e. it was different in 50% of the trials), by pressing one of the two predefined keys on the keyboard after the removal of the second stimulus. Response time was not limited and subjects had to respond to proceed to the next trial. The next trial began after either the subjects responded or after 800 ms post-stimulus onset whichever happened later. Subjects seated in a dimmed room 60 cm away and against an Asus VG24QE monitor on which the visual stimuli were presented. Matlab PsychToolbox^37^ was used for designing the task, presenting the images and recording the responses. Objects’ images covered between 2.5 to 13.5 degrees of visual angle depending on their size conditions. Each unique object image (i.e. 192 images in the image set) was presented to each subject three times in random order (adding up to 576 stimuli). Specifically, each subject was presented with a randomly-ordered presentation of three repetitions of the same 192 images in the dataset (images were presented randomly as the first or second image in each trial). The repetition of presentation was aimed at increasing the signal to noise ratio in the analyses. Trials were divided into three blocks with five minutes of resting time between the blocks. Subjects participated in a short training session before the main experiment on a different image set to get acquainted with the task.

Two major considerations were made when designing the paradigm to avoid interfering factors in the results: (a) the paradigm was designed in passive format (i.e. subjects performed an irrelevant task and did not actively categorize objects) to avoid the involvement of top-down cognitive processes such as attention and expectation (as they may modulate the dynamics of visual processing in the brain^34,35,38^) and allow only the sensory object processing mechanisms to function; (b) images were presented very shortly (i.e. for only 50 ms) to avoid the domination of feed-forward information in the recorded signals to be able to dissociate between feed-forward and feedback flows of information^8,19,23^.

### Participants

Ten human subjects (average age 22 years, three females) volunteered for this single-session EEG recording experiment which lasted for about 45 minutes. Subjects had normal or corrected to normal vision. Informed consent was obtained from every participant. All experimental protocols were approved by the ethical committee of Shahid Rajaee Teacher Training University. All experiments were carried out in accordance with the guidelines of the declaration of Helsinki and the ethical committee of Shahid Rajaee Teacher Training University.

### Signal recording and preprocessing

A 32-channel eWave32 amplifier was used for signal recording which followed the 10–20 convention of electrode placement on the scalp (see Supplementary Fig. S1B for electrode locations). The amplifier, produced by ScienceBeam (http://www.sciencebeam.com/), provided a sampling rate of 1 K samples/second which allowed me to investigate the temporal dynamics of information processing in the brain very accurately. The recorded data was taken into Matlab (http://www.mathworks.com/) and all the following analyses were performed using custom codes in Matlab. I band-pass filtered the recorded signals in the range between 0.5 to 100 Hz to filter-out the DC component as well as the high-frequency noise. I also notch filtered the signals at 50 Hz to block the line noise. In order to remove eye-blink, body and eye movement artifacts, Independent Component Analysis was used as implemented by the EEGLAB toolbox^39^. The filters were finite FIR filters (12 dB per octave roll-off) and the ICA artifact removal used the *runica* algorithm^39^. The ADJUST plugin^40^ was used for determining the artefactual components, which statistically evaluated ICA components and suggested the components which contained the mentioned artifacts for removal. An average of 3.8 components (min = 2 and max = 6) were removed from the analysis for each subject. A total of 154 trials (mean = 2.67%, sd = 1.4%) were also removed from the total set of trials from all subjects as they were diagnosed to be artefactual by visual inspection. Signals were then broken into epochs (i.e. analysis time windows) which were aligned to the stimulus-onset, in the range from 200 ms pre- to 800 ms post-stimulus onset. Signals were then smoothened using a 5-sample non-overlapping moving average filter to attenuate the spurious patterns in the signals. The resulted signals were used in the representational analyses.

### Representational analysis of patterns

For each subject, after the preprocessing steps, an ‘X’ data matrix was constructed which included activity values (i.e. voltage values in microvolts) obtained from electrodes. X was a 3-dimensional (31 × 201 × 576) matrix incorporating signals from every one of the 31 electrodes (one electrode was the reference electrode and was put on the right mastoid), at every 5 ms time point (obtained in the range from 200 ms pre- to 800 ms post-stimulus onset resulting in 201 time points) across every one of the 576 trials (assuming that the subject had no removed trials). The representational analysis was performed on the signals obtained from both the first and second stimuli in the trials.

The representational analysis method of the current study has been previously explained in full details^36,41^. Briefly, I report a decodability index referred to as d′ (i.e. which has also been used to measure sensitivity, separability, selectivity and discriminability in previous studies^42–44^) to show how separable the clusters of distinct conditions (either category or variation conditions) positioned relative to each other in the brain space and how their distributions changed over time (Supplementary Fig. S1A). This decodability measure is advantageous to classification-based decoding methods as it is robust when data clusters include unequal samples^44^ (e.g. the analysis of ‘variation processing’ in the current study.

**Figure 2:**
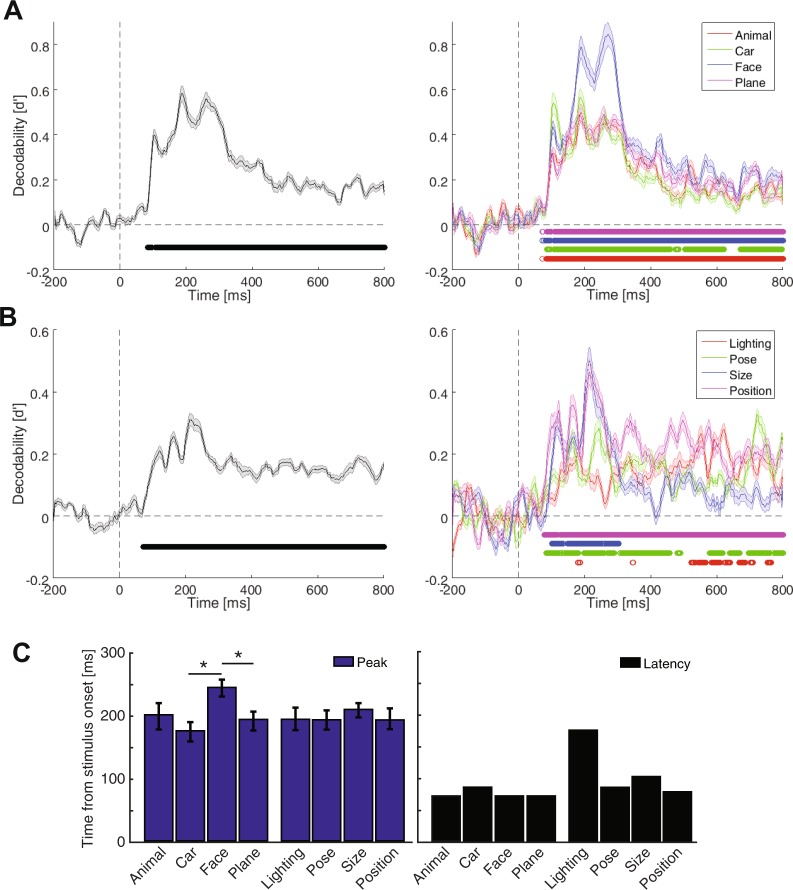
Time-resolved decodability of categories (**A**) variation conditions (**B**) and their temporal statistics (**C**) for the pooled-condition case. Left columns in (**A** and **B**) show category- and variation-pooled results and right columns show the same results resolved into constituent categories (**A**) and variations. (**B**) The vertical and horizontal dashed lines indicate respectively the stimulus onset time and the zero decodability value. The circles indicate the time points at which the color-matched decodability curve was significantly above the decodability values averaged in the last 200 ms pre-stimulus (i.e. p < 0.05, Wilcoxon’s signed-rank test). (**C**) Latency (black) and peak (blue) time points of decoding in category and variation decoding decodability. Stars show significant (p < 0.05, Wilcoxon’s signed-rank test) difference between peak time bars. Shaded areas and error bars indicate the SEM across subjects.

**Figure 3:**
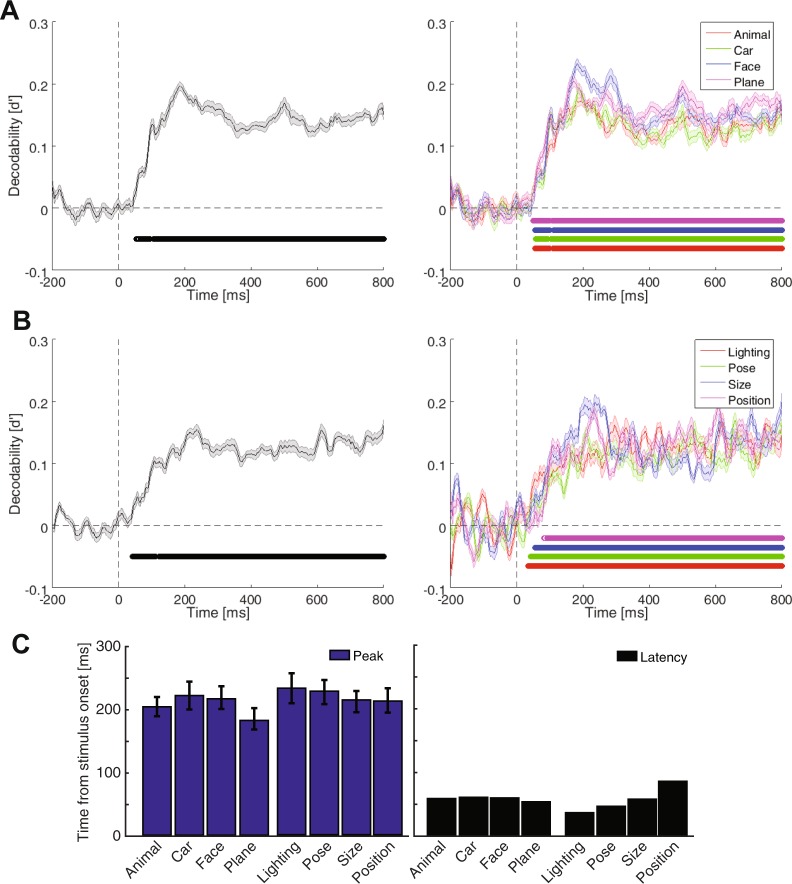
Time-resolved decodability of categories (**A**) variation conditions (**B**) and their temporal statistics (**C**) in the per-condition case. All the details are the same as in Fig. 2.

**Figure 4:**
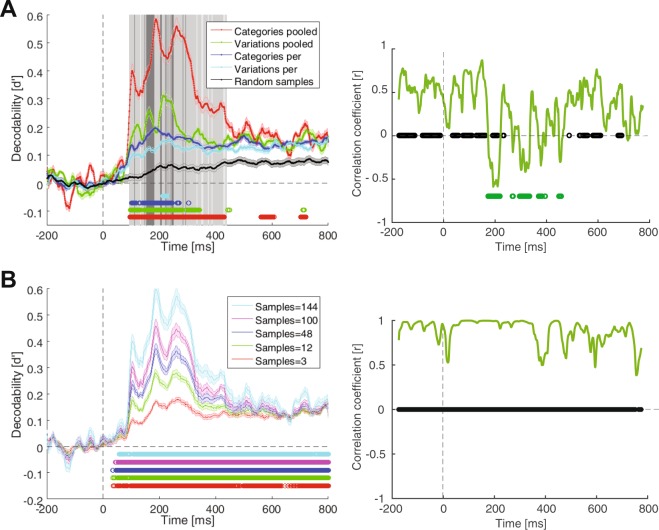
Comparison of time-resolved decodability of categories and variations in the pooled- and per-condition cases. (**A**) Left, the black decodability curve shows the results of random sub-sampling of the whole stimulus set. The vertical solid light and dark gray lines indicate the time points at which respectively the category and variation decodability curves showed significantly (p < 0.05, Wilcoxon’s signed-rank test) higher pooled- than per-condition values. The colored circles indicate the time points at which the corresponding decodability curve showed a significantly (p < 0.05, Wilcoxon’s signed-rank test) higher value compared to the randomly sub-sampled black curve. Shaded areas indicate the SEM across subjects. (**A**) Right, the Time-resolved correlation between per-condition cases of category and variation decodability curves. (**B**) Sub-sampled category decodability curves for different number of samples (left) and the correlation values between the 144 and 3-sample curves (right). The black circles indicate time points of significant correlations (p < 0.05, Pearson linear correlation, corrected for multiple comparison across time points) and the green circles indicate time points at which correlations were significantly different from average of correlations in the last 200 ms window prior to stimulus onset (p < 0.05, Wilcoxon’s signed-rank test).

In the remaining of this section, I will explain the procedure of representational analysis using an example for clarification. In order to calculate the decodability of conditions across car and face categories at 150 ms post-stimulus time point in the recorded EEG space, I used the data from all 31 rows of the 71^th^ column of the ‘X’ data matrix. The data included the matrix’s trials 1 to 144 which represented the car data as well as trials 145 to 288 which contained the face data. The mentioned car and face data formed two category clusters in the electrode (representational) space (Supplementary Fig. S1A). The clusters provided a pair of 31-dimensional mean vectors which were used for dimension reduction to simplify the reporting of clusters’ decodability in the representational (brain) space. In order to reduce the dimension of the representational space from 31 to one, the cluster data points were projected onto the line connecting the two clusters’ means. Accordingly, a pair of 1-dimensional mean and a pair of 1-dimensional variance values were obtained from the two clusters which were used to calculate the decodability value (d′) using (1):

1 $$d^{\prime} =\frac{{\mu }_{1}-{\mu }_{2}}{\sqrt{\frac{1}{2}({\sigma }_{1}^{2}+{\sigma }_{2}^{2})}},\,{\mu }_{1} > {\mu }_{2}$$

where *μ*s and *σ*s are the mean and variance values obtained from the two clusters (i.e. categories in the above example) and *d*′ represents the separability/decodability value between the face and car categories. Note that, according to eq. (1), the cluster with a higher mean voltage should always be considered as cluster ‘#1’ to result in positive d′ values, which is actually a distance value and should always be positive. The d′ value was calculated for every time point to obtain the time-resolved results depicted throughout the paper (e.g. Fig. 2). For the average decodability curves (Fig. 2A, left and 2B, left), the decodability indices were calculated for and averaged across all possible pairs of conditions (e.g. between car and animal, car and plane, etc.). The average decodability value (Figs 2 and 3, right) for the car category was obtained by averaging all three decodability curves in which the separability of the car category was measured from other categories. The baseline decodability value (i.e. calculated as the average decodability value within the last 200 ms pre-stimulus window) was subtracted from the corresponding decodability values (within all pre- and post-stimulus time points) leading to mainly positive decodability values across the curves. The representational analysis procedure was repeated for every subject to obtain individual’s decodability results, before they were averaged to provide across-subject averaged results (Figs 2–4, shaded error areas represent standard error across subjects). Other decoding methods (i.e. SVM and LDA classifiers) were also tested on the recorded data and provided similar results.

### Analysis of Granger causality

In order to investigate the transactions of category and variation information between peri-occipital and peri-frontal areas, a recently proposed version of Granger causality analysis was used^19^. The logic behind Granger causality is that time series Y might have caused time series Z if Y contains information that facilitates the prediction of future values of Z compared to when considering the information in the past of Z alone. As an example, assume the case of category information moving from posterior to anterior brain areas. In this case, it can be concluded that category information has moved from peri-occipital areas and reached peri-frontal areas if the past representations of peri-frontal alone are not as predictive of the current category representations on the peri-frontal areas as the past representations of peri-frontal plus past representations of peri-occipital are.

First, I needed to have obtained object representations to be able to follow their movement on the scalp. For that purpose, I used the well-known similarity matrices^45^, which can provide cross-correlation values between pairs of representations. These matrices contain similarity/dissimilarity indices (i.e. indices can be measured using Euclidean distance, correlation coefficient, etc.) calculated across the representations of stimulus pairs (i.e. stimuli can be from the same category in the case of within category analysis, or across variation conditions in the case of variation analysis). Here, the similarity matrices contained correlation coefficients (obtained by Pearson linear correlation) across pairs of 9-dimensional brain representations (as obtained from nine frontal/occipital electrodes) of categories and variations. The dimension of representational space was determined to include a subset of electrodes to separate the peri-frontal from peri-occipital representations. Therefore, two similarity matrices were obtained at each time point; one from the peri-frontal (including F3, F4, F7, F8, FZ, AFZ, FP1, FP2, FPZ) and one from the peri-occipital electrodes (including P3,PF4, P7, P8, PZ, POZ, O1, O2, OZ) (Supplementary Fig. S1B). For example, in order to obtain the similarity matrices of categories, on a single variation condition (e.g. first size condition) at each time point, a 48 by 48 similarity matrix was constructed which included correlation coefficients between all possible pairs of the 16 objects (Fig. 1A, each unique image was presented three times during the experiment). The symmetric sides of the similarity matrices (the top right side which contained values similar to the symmetric bottom left cells) as well as their diagonal axes were excluded from Granger analysis. According to Goddard *et al*.^19^, partial correlations were used to calculate a simplified version of Granger causality. Equations (2) and (3) provide feed-forward as well as feedback flows of information on the brain at every time point:

2 $$FF(t)=\rho S{M}_{(front,t)}S{M}_{(back,t \mbox{-} past)}.S{M}_{(front,t \mbox{-} past)}$$

3 $$FB(t)=\rho S{M}_{(back,t)}S{M}_{(front,t \mbox{-} past)}.S{M}_{(back,t \mbox{-} past)}$$

where *SM*_(*loc*,*t*)_ is the similarity matrix obtained from location *loc* at time *t* post-stimulus onset, and *SM*_(*loc*,*t−past*)_ is the similarity matrix which was obtained by averaging the similarity matrices in the window from *t* − 130 to *t* − 80 ms post-stimulus onset on the same location. The rationale behind choosing the mentioned time window was that, it was covered by the range from 72 to 141 ms which was previously shown to reflect the time span during which occipital to prefrontal flow of information was observed^46^.

### Statistical testing

To evaluate the significance in differences between the peaks of decodability curves across subjects (e.g. Fig. 2C), Wilcoxon’s signed-rank test was used. In order to evaluate the significance of decodability and information-selectivity indices (e.g. Figs 2–4) at a post-stimulus time point, I evaluated the vector of post-stimulus decodability indices (including ten decodability values corresponding to ten subjects) against their respective values averaged in the last 200 ms pre-stimulus window prior to baseline removing, using Wilcoxon’s signed-rank test.

In order to evaluate the significance of the partial correlation values at each time point in the Granger causality analysis, a null distribution of correlation values was generated at each time point by shuffling the elements of the similarity matrices and then using the scrambled matrices in eqs (2 and 3). One thousand random correlation values were generated at each time point by repeating the shuffling procedure and calculating the random correlations, against which the true correlation values were assessed for significance. A correlation value was considered significant if it surpassed 95% (i.e. 950) of the randomly generated correlation values.

**Figure 5:**
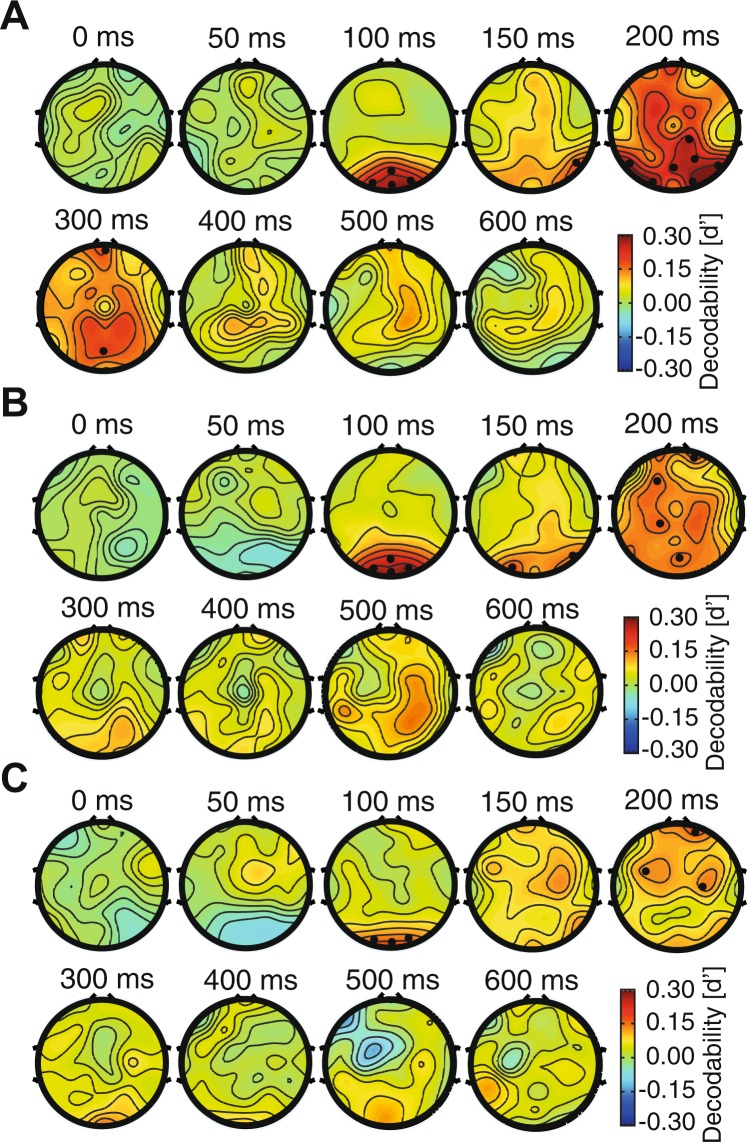
Scalp maps for category and variation decodability. These maps were generated by measuring the decodability indices for each electrode separately and finally using their superposition on the scalp. The decodability values between the electrodes were calculated by interpolation as implemented in EEGLAB. (**A**) Pooled-condition category decodability maps. (**B**) and (**C**) Per-condition category and variation decodability maps at specific time points. The reported decodability values are averaged in the span from −25 m to +25 ms relative to the indicated time points. The dots show electrodes with significantly higher decodability values compared to the last 200 ms prior to stimulus onset (as evaluated with Wilcoxon’s signed rank test with correction for multiple comparisons).

**Figure 6:**
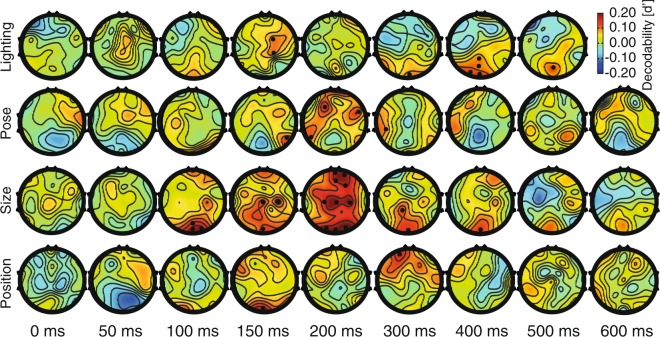
Scalp decodability maps separated for each variation. From top to bottom, decodability maps are provided across conditions of lighting, pose, size and position, respectively. Other details are the same as in Fig. 5.

The results of statistical tests (e.g. Wilcoxon’s signed-rank test) as well as linear/partial correlation values were FDR-corrected (using Matlab *mafdr* function) for multiple comparisons throughout the analyses wherever multiple time points were tested simultaneously. The *mafdr* function received *n* p-values obtained from *n* statistical tests (*n* is the number of comparisons or time points) and provided as the output, the same number of p-values which have been corrected for multiple comparisons. The multiple comparison correction of the electrodes on the topographic maps (Figs 5 and 6) were done in the same way as was done for the multiple time points with *n* representing the number of p-values obtained from individual electrodes on each of the related maps in the series (31 × 9). The significance threshold was 0.05 in all the analyses.

### Computational model

In order to see if a hierarchically organized model of human object processing could provide an explanation for the observed representational mechanisms in the brain, the image set was also fed to a recently developed model of human object recognition. The model known as ‘AlexNet’ has been shown to closely replicate object representations obtained from higher visual areas of the human and non-human primates’ brain^47–49^. I used the model’s Matlab implementation^50^ which was freely available at (http://www.vlfeat.org/matconvnet/). As the model has been previously explained in many studies^8,47,48^, extra explanations are avoided here. Briefly, the model is an eight-layer convolutional neural network which has been trained on a set of 1000 object categories from the ImageNet Large Scale Visual Categorization (ILSVRC; http://www.image-net.org/)^51^, including the categories used in the current study, using gradient descent algorithm. The model utilized several mathematical operations such as convolution, maximization, normalization and pooling in alternating layers. The first five layers of the model implemented convolutional operations which were followed by three layers of fully-connected units. Since the last layer worked as a 1000-class classifier in previous studies^50^, I used layers one to seven in the current study. Each output unit at each layer was treated as a representational dimension (i.e. corresponding to EEG channels) and decodability indices were obtained from each layer’s output according to eq. (1).

## Results

This study was designed to provide spatiotemporal insight into object category and variation processing at the whole-brain scale. For that purpose, ten human subjects participated in a passive EEG recording paradigm in which they reported if the fixation spots, which accompanied two consecutively presented objects, were the same color or different in each trial. Subjects performed the color-matching task with significantly above-chance accuracy (mean = 95.75%, std = 4.03%, p < 0.001, Wilcoxon’s signed-rank test) and in reasonable time (mean response time = 729 ms, std = 97 ms) meaning that they were alert and attentive to the task during the experiment.

### Temporal dynamics of category and variation processing

To investigate the temporal dynamics of category and variation processing in the brain, I calculated decodability indices (referred to as information in the text) across categories and variation conditions (Fig. 2A and B). The category information (Fig. 2A, left; averaged across all possible pairs of categories) showed a highly dynamical pattern; it rose to significance at 84 ms, showed three peaks (with the highest peak at 184 ms) and remained significant until 800 ms post-stimulus onset. Interestingly, the variation information rose to significance at 71 ms post stimulus, earlier than the category information. Although indicating lower peaks compared to category information, the variation information (Fig. 2B, left, averaged across all four variations) revealed a similar pattern showing three peaks with the highest peak at 214 ms post-stimulus. It remained significant until the last analysis time point at 800 ms.

In order to obtain insight into the possible differences between different categories and variations, I resolved the averaged results (Fig. 2A and B, left) into their constituent categories and variations (Fig. 2A and B, right). Although all four categories experienced the same three peaks (Fig. 2A, right), higher decodability values were observed for the car category during the first peak (which was at 105 ms post-stimulus) which was dominated by the face information during the second and third peaks (which occurred respectively at 188 ms and 271 ms post-stimulus). It should be noted that, the reported category information values were calculated between pairs of categories; therefore, a higher information value for face means, when comparing its separability from the other three categories, faces representations positioned more separately compared to how every other category representations positioned relative to the rest of categories. For detailed across-category information plots see Supplementary Fig. S2A. While rising to significance at an earlier time point compared to the other categories (at 71 ms), the face category also peaked at a significantly later time (mean = 243 ms) compared to car and plane categories (Fig. 2C, left, p < 0.05, Wilcoxon’s signed rank test). Decodability curves showed undistinguishable latencies across categories which ranged from 71 to 85 ms post-stimulus (Fig. 2C, right). Latency was defined as the time distance from stimulus onset to the first time point at which the decodability indices rose to significance. The appearance of face information in the late peaks of category information was explainable by the N170 component of ERP signals which have often been associated with the processing of faces in the brain^52^. The previously suggested precedence of intermediate-level (e.g. face-plane and car-face) to subordinate-level (e.g. car-plane) and superordinate-level (e.g. animal-plane and animal-car) category information was also noticeable in the temporal dynamics of category information^53^ (Supplementary Fig. S2A).

I also evaluated the decodability of variation conditions in the signals. The first goal of this study was to see whether information about different variations could be extracted from human brain signals. The temporal dynamics of position information processing has been previously studied and showed close relationships with category-related information^28^, however, the processing of other variations have remained overlooked which is addressed by the following analyses.

Within-variation decodability results which were averaged across all pairs of conditions showed that, while conditions of all variations were decodable from brain signals, the conditions of position and size were more distinguishable than the conditions of pose and lighting (Fig. 2B). It means that information about different object sizes and positions were easier to differentiate compared to the conditions of the other two variations from brain signals. This difference could not be explained by the pixel-space decodability indices calculated on the image set in the pixel space which showed information values (d′) of 2.95, 1.63, 1.44 and 2.2 for lighting, pose, size and position variations, respectively. Therefore, it seems that, not all variations were processed similarly by brain mechanisms. This can be explained by both the difference between the scale of cortical coverage across different variations’ conditions (i.e. which can enhance the multi-variate decodability of variation conditions of size and position which involved larger retinotopic cortical areas compared to lighting and pose) and the difference in the processing mechanisms of the brain in the compensation of different variations^8,29,49^. The latter reason can also be supported by the category decodability indices obtained under each of these variations (Supplementary Fig. S2C), with lighting and position respectively causing the lowest and highest degrees of degradation of category decodability. Lighting rose to significance much later than the other variations, but none of the variations showed a significantly different peak time (Fig. 2C, left). It should be noted that the analysis of variation decodability, which unveils the dynamics of variation processing in the brain, is different from the decoding of categories under variations which aims at comparing the impact of variations on category-related information processing, also known as invariant object processing^8,54^.

A previous study named the non-categorical object-accompanying information (i.e. variations), the ‘category-orthogonal properties’, implying that these information might be processed by mechanisms which are not necessarily involved in the processing of object categories^23^. Yet, in the above analyses, when calculating the category information, the data from all variation conditions were included in the representational analysis. Moreover, when calculating the across-condition information of different variations, the data from different category exemplars were considered in the representational analysis. I thought that, this might have influenced the above results by allowing the interaction of category and variation information in the analyses, as it might have been the case in previous studies^25,54^. More specifically, in the case of category decodability, each of the observed peaks in the category decodability curves (Fig. 2A) could have been evoked by either the dynamics of category processing or the repositioning of data points caused by the processing of variations in the representational space, which could have led to the enhancement of category information. Therefore, in order to avoid the interaction between category and variation information in representational analysis, I calculated the category decodability indices on every single variation condition (to obtain category information) and calculated the variation decodability indices on every category exemplar (to obtain variation information) before finally averaging them (Fig. 3). I will call these new analyses as ‘per-condition’ and the former analyses as ‘pooled-condition’ in the following sections. Although the decodability curves lacked the large bump of information, which happened before 300 ms post-stimulus onset and dominated the later decodability values, compared to the pooled-condition case (compare Figs 2 and 3), the results repeated many of the observations from the pooled-condition analysis. The average category and variation information (Fig. 3A and B, left column) rose to significance respectively at 53 and 41 ms, showed three bumps in the first 300 ms and remained significantly positive until 800 ms post-stimulus. The dominance of face and size information could also be observed. Neither the category nor the variation information showed significantly different peak times across their constituent conditions (Fig. 3C, left). The ranking of pairs of category information remained almost intact (compare Supplementary Fig. S2A and B) and lighting still provided the least amount of influence on category information (compare Supplementary Fig. S2C and D). See also Supplementary Fig. S3A for the results within each variation in the per-condition case. Therefore, it seems that little influence was imposed by the variation information when decoding category information and vice versa. This is investigated more thoroughly in the following sections.

### Temporal relationship between category and variation processing

As many previous studies have investigated the decodability of category information from brain activities^28,54,55^, two goals were pursued in this study: to investigate the spatiotemporal dynamics of variation processing, and to assess the spatiotemporal interaction between variation and category processing in the brain.

In order to address these questions, I had to first choose either the per- or pooled-condition decodability results for the following analyses. Qualitative comparison between per- and pooled-condition results (Figs 2 and 3), done in the previous section, supported that their main differences were observed in the early time windows. In order to provide a more accurate insight into the temporal pattern of category and variation processing in per- and pooled-condition cases, I provided a summary of the above-mentioned results on a single plot (Fig. 4A, left). As obvious from the curves, the three bumps of information occurred at around the same time in the per- and pooled-condition cases of category processing. This proposes that, as the per-condition decodability curve was obtained on single variation conditions (therefore not influenced by other variation conditions) and showed the same three bumps, the information on the pooled-condition category curves were majorly driven by category information rather than by variation-related processing. After investigating the per- and pooled-condition curves of variation decodability, the same conclusion could be made about the variation information, supporting minor influence of category information on variation processing. In order to highlight the main time spans during which the per- and pooled-condition cases differed, I indicated the time points at which the information values were significantly higher in the pooled- compared to the per-condition deocodability curves using light and dark gray vertical lines respectively for the category and variation information. Accordingly, the first and the last time points at which the category (and variation) information were significantly higher in the pooled- compared to per-condition were respectively 92 ms (and 112 ms) and 438 ms (and 294 ms). These results are on par with the reported window of sensory visual processing in the human brain^55,56^ suggesting that the higher number of samples in the pooled compared to the per-condition case in decoding, affected the window of sensory visual processing rather than the later windows of processing which are generally associated with higher order cognitive processes. Despite their similarities, I used the per-condition case of decoding to obtain the results provided in the following sections (all results after Fig. 5A) of the paper to avoid unnoticed interactions between category and variation processing.

Before using the per-condition decodability curves in the following analyses, I had to determine if the calculated decodability indices were significantly above chance decodability values which could be obtained from a randomly labeled dataset. This could determine the level of baseline category-unrelated information in the dataset which could have contributed to the reported decodability curves. Therefore, I assessed the four mentioned decodability curves against a decodability curve obtained from 144-sample randomly chosen stimulus sets (Fig. 4A, left, the black decodability curve). It can be observed that the four curves (even the per-condition curves of categories and variations which consisted of much fewer stimuli) showed significantly above-chance decodability values (as indicated by color circles). The level of significance was very hard to achieve for the per-condition variation decodability curve as it included only three data points within each data cluster in the representational analysis compared to the random sub-sample which consisted of 144 data points within each cluster. In order to generate the mentioned random decodability curve, I randomly selected a subset of 144 stimuli from the whole set of 576 stimuli of each subject (ignoring the category and variation labels of the chosen stimuli) and repeated the random representational analysis 1000 times before averaging them on each subject. Together, these results support that the decodability curves, even in the case of per-condition analysis, contained category information which significantly surpassed the information in any randomly chosen subsample of the data which could have provided information contributing to the reported category and variation information.

After ensuring that the brain signals contained significant amounts of category and variation information, I approached the first question of the study by comparing the temporal dynamics of category and variation information. Examining the temporal dynamics of category and variation processing curves (in Fig. 4A, left) suggested that these curves did not follow the same temporal pattern (e.g. compare their peaks and valleys at around 200 ms post-stimulus). In order to provide a quantitative comparison, I evaluated the time-resolved correlation between category and variation information curves in their per-condition cases (i.e. no noticeable difference was observed when I analyzed pooled-condition cases). Specifically, I calculated the correlation coefficient (Pearson linear correlation) between the category and variation decodability time series within the same 50 ms sliding time windows across time (Fig. 4A, right). Correlations were considered significant if their *p*-value was smaller than 0.05 (after multiple comparison correction) and therefore the corresponding significant time point were indicated on the time axis by black circles. Interestingly, while showing a rising correlation trend from the stimulus-onset to around 150 ms, and a falling trend after around 580 ms, the correlation coefficient curve experienced several systematically negative windows in the span from 173 to 464 ms post-stimulus. Significantly negative correlations were observed during the 192 to 212 ms post-stimulus window. These results suggested that, after the stimulus onset, three stages of visual processing in the temporal pattern of category/variation processing could be observed in the brain: a first stage which started after the stimulus onset time and ended at around 170 ms in which information about category and variation was processed in in-phase patterns; a second stage which started at around 173 ms and ended at around 450 ms in which categories and variations underwent several anti-phase processing time spans and a third stage which started at around 470 ms and was observed until the end of visual processing with in-phase processing of categories and variations. This suggestion will be supported by further analyses in the following sections.

It was suspicious that the observed difference between the phases of category and variation processing might have been caused by the difference in the number of samples (i.e. number of representational points obtained by stimulus presentations) considered when comparing category with variation processing. The number of representational points were 144 (and 48) in the pooled and 12 (and 3) in the per-condition cases of category (and variation) conditions in the decodability analysis. To check if this was the case, I down-sampled the stimulus set used in the representational analysis of category data clusters from 144 to 100, 48, 12 and 3 (Fig. 4B, left) and re-calculated the correlations between all possible pairs of subsets, but no negative correlation was observed at any time point (i.e. the sampling procedure was repeated 100 times and the results were averaged before being compared to the true 144-sample decodability curve). In other words, I used a subset of stimuli in this representational analysis. Results of correlations between the 144- and 3-sample subsets, as the most distant cases, are shown in Fig. 4B, right. Therefore, the time-dependent phasic dynamics between category and variation processing seems to be inherent in the brain, and not an effect of the number of samples used in the analyses.

### Spatial dynamics of category and variation processing

In order to compare the contribution of different brain regions to the processing of categories and variations, I calculated the decodability indices on the scalp using a univariate methodology (Fig. 5). For that purpose, as opposed to the above results which were obtained from all the 31 scalp electrodes, here I report single-channel decodability indices on time-specific scalp maps^55^. In other words, instead of in 31-dimensional space, the representations were evaluated in a one-dimensional space. Please note that the decodability indices were interpolated to find decodability values in areas between electrodes using EEGLAB. Figure 5A shows the pooled-condition category decodability results on the scalp, which has been the most common type of category information reported previously, which includes both category and variations^55^. The amplitudes of decodability indices are lower here compared to those reported in the 31-dimensional space (Figs 2–4), as a result of dimension reduction in the representational space from 31 to one. The reported decodability values are the average of the decodability indices obtained in the time-window from 25 ms before to 25 ms after the indicated time instances. Above-baseline category information was observed in the 50 ms as well as 100 ms windows at the AFZ and FZ electrodes with significantly (p < 0.05, Wilcoxon’s signed-rank test) above-baseline information at 100 ms in the posterior brain (POZ, O1, O2 and OZ). See the statistical testing section for the details of statistical testing procedures. In the 150 ms and 200 ms windows, significant category information was observed on occipitotemporal (O1, O2, P7 and P8), parieto-central (P4, POZ and CP2) with high but non-significant frontal (F3, F4, FZ, AFZ, FPZ, FP1 and FP2) areas. At 300 ms post-stimulus onset, parietal (P3, P4, POZ (p < 0.05) and PZ), central (C4) and frontal areas (FC2, FZ, F4, AFZ and FPZ) showed category information which declined in the following time windows (i.e. 400, 500 and 600 ms). Together, these patterns of distribution repeated many previous observations, which have reported the involvement of occipital, occipitotemporal, parietal as well as frontal areas, in category processing following similar temporal dynamics^1,25,55^. However, as explained earlier, their reported category information may have been influenced by the information from variations. Therefore, I also provided the per-condition category (Fig. 5B) and variation (Fig. 5C) processing scalp maps. Although noisier here compared to the pooled-condition results, the three initial windows (i.e. 0, 50 and 100 ms) of both categories and variations repeated the pooled results. While the category information was more concentrated on posterior electrodes (O1, O2, OZ, P8, CP1 and POZ) plus several frontal electrodes (F3 and FP2), variation information was found mainly on frontal (FC2, FC5 and FPZ) areas in the 150 and 200 ms windows. Parietal information (i.e. information averaged across P3, PZ and POZ electrodes) was significantly (p < 0.05, Wilcoxon’s signed rank test) higher for categories compared to variations in the 200 ms window. In the following windows (300–600 ms), category information was observed in both parietal and frontal areas, while variation information was processed dominantly in occipital and frontal regions. These results which presented separated category and variation information on the scalp, showed evidence supporting both spatiotemporally shared (in the 100 ms window between category and variation processing maps) as well as distinct (in the 200 ms window) mechanisms involved in the processing of category and category-orthogonal properties (i.e. variations).

It has been previously suggested that all variations are not necessarily processed by the same set of brain mechanisms in object recognition. In other words, it has been suggested that while size and position are processed by the feed-forward mechanisms of the ventral visual stream, variations such as pose and lighting may need top-down feedback signals from higher cognitive areas such as prefrontal cortex to be compensated for during recognition^8,29,32,49^. To investigate this, I plotted the scalp maps of each variation separately (Fig. 6). In the 50 ms window, information regarding all variations could be found in the centro-frontal (FC1, FC2, F3, F4, F7, F8 and FZ) brain areas significantly (p < 0.05, Wilcoxon’s signed-rank test) easier than it could be found on occipital (O1, O2 and OZ) and parietal (PZ and POZ) areas. In the 100 ms window, information regarding all variations could be consistently found on occipital areas (O1, O2 and OZ). In the 150 ms window, while the information regarding lighting and pose could be observed mainly on centro-frontal areas (CZ, FC1, FC2, F4, F8, FZ and AFZ) and not in occipitotemporal areas (O1, O2 and OZ), size and position information were mainly concentrated in occipitotemporal areas (O1, O2 and OZ). Almost all variations showed a frontal concentration in the 200 ms window with higher values for variation in size (which is probably explained by larger stimuli which evoked higher brain responses). Pose conditions exposed the previously proposed co-activation of peri-frontal (F3, F4, FZ, AFZ, FP1, FP2 and FPZ) and occipitotemporal areas (P3, P4, P7 and P8; Serre *et al*.^29^), which may suggest the feedback of pose information from PFC to IT cortex. Lighting information consistently covered the occipito-parietal areas in the subsequent windows (from 300–500 ms). During the same windows, pose information was mainly found on temporal as well as frontal areas enhancing the mentioned possibility for the interaction of those areas. Interestingly, size information lingered on occipito-parietal areas (O1, O2, OZ and POZ) as well as frontal (F8 and FP2) areas which might be explained by previously suggested frame-transformation in object processing performed in parietal areas^57^. As previously observed by several studies^8,28,54^, position information showed a late appearance in the 300 ms window (see also Fig. 3B, right), and appeared on specific temporal (T7) and frontal areas (F4). By showing that not all variations exposed similar brain patterns, it could be supported that some variations could have activated auxiliary mechanisms such as feedback signals from higher cognitive areas of the brain. However, a quantitative evaluation was needed to reveal whether variations and categories were processed by the same brain mechanisms and whether there was any interaction between peri-occipital and peri-frontal areas regarding these processes. In the following sections, these concerns are addressed.

### Selectivity for category and variation information in the brain

**Figure 7:**
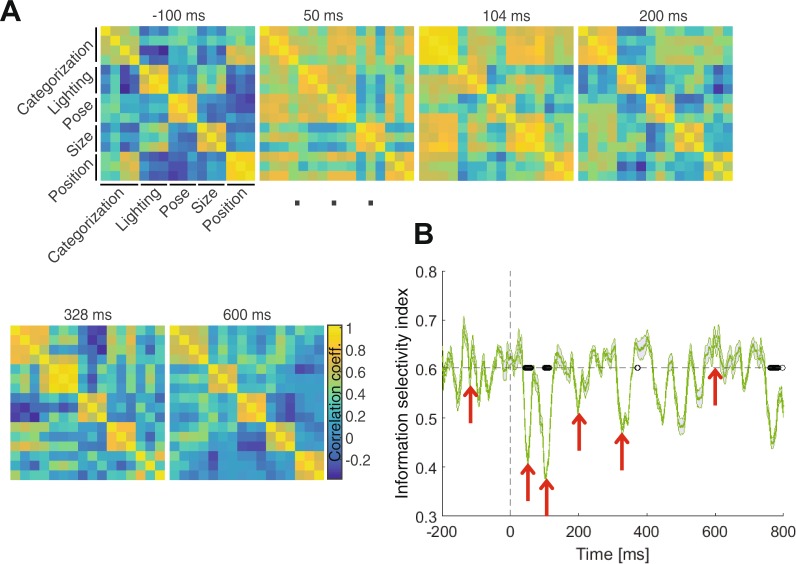
Information-selectivity in different brain areas. (**A**) Information-selectivity matrices showing, in color codes and at specific time points relative to stimulus onset, the entangling of category and variation processing in the brain. Colors show the amount of correlation (Pearson linear correlation) between decodability indices obtained from whole-brain EEG electrodes in the decoding of specific aspects of information with higher values showing more similarity (information non-selectivity). (**B**) Time-resolved information-selectivity, measured as the difference between the sums of within-information correlations minus the sum of across-information correlations. Red arrows indicate the time points used in (**A**). The black circles indicate the time points at which the information-selectivity index was significantly (i.e. p < 0.05, evaluated using Wilcoxon’s signed-rank test) different from the same index averaged in the last 200 ms window prior to the stimulus onset (before baseline removal). Shaded areas indicate the SEM across subjects.

In order to quantitatively determine whether categories and variations were processed by the same neural structures, I employed a recently-proposed methodology which was developed and used to evaluate the role of single neurons in the processing of categories and several variations by measuring their selectivity for each of these processes^23^. I replaced the selectivity indices used in that paper^23^ by the decodability indices obtained from individual electrodes here. More specifically, to know how information-selective different brain areas were, I constructed information-selectivity matrices (which the original study called task-specificity matrices^23^) that reflected color-coded correlation coefficients within and between information dimensions (i.e. category- and variation-related aspects of information) at several key time instances (Fig. 7A). Please note that, by ‘selectivity’ I mean the tendency of individual brain areas in the processing of specific types of information (i.e. category- and variation-related information). More specifically, if an electrode could differentiate between different categories (e.g. showed stronger activity for animals compared to cars) the selectivity of the area under that electrode would be for discrimination of animals from cars and if it provided discriminable activities for two lighting conditions its selectivity would be for the encoding of the two lighting conditions, or if showed both types of encoding, it was considered selective for both aspects of information. It should be noted that an area can encode unlimited aspects of information (i.e. not necessarily covered in this study). Therefore, in order to evaluate the information-selectivity on the whole-brain scale, the amount of correlation (Pearson’s linear correlation) was evaluated at each time point, between the decodability indices found for the vector of 31 electrodes on one aspect of information (e.g. decodability of animal from other categories) and another (e.g. decodability of size’s first condition against other conditions). Accordingly, if the set of 31 electrodes provided similar (correlated) decodability patterns in the 31-dimensional space across two aspects of information, the correlation coefficient would be close to unity meaning that the two aspects were encoded (processed) by the same set of electrodes (and possibly the same mechanisms as the correlations were measured on millisecond time scale).

The information-selectivity matrices showed a high-level of information-selectivity at −100 ms prior to stimulus-onset (Fig. 7A). Although instances of confusion between some aspects of information (e.g. between categorization and position processing, between size and lighting processing, etc.) could be observed, higher values of within-aspect correlations could be observed compared to between-aspect correlations. This is not surprising since the information-selectivity is not related to the amplitudes of the decodability indices, which are naturally low in the pre-stimulus span, but rather to the decodability patterns (i.e. whether high or low) across electrodes, therefore unrelated to the presentation of the stimulus. In other words, information-selectivity is another way of looking at ‘background connectivity’ in the brain, which is defined within/across neural populations as the involvement of different neural populations in same/different information processing aspects^58,59^. This background connectivity, which reflects inherent in the brain, is independent of the amount of input information (i.e. stimulus presentation)^58,59^. Therefore, it is not strange to observe a high level of information-selectivity in the pre-stimulus span as this pattern can also be observed in the very late processing time points (e.g. at 328 and 600 ms post-stimulus) during which the input stimulus has almost no effect. Here, however, I concentrated on time instances of significant drops of information-selectivity which reflected the co-processing (entangling) of information aspects in shared brain areas. The processing of different aspects of information have been totally entangled with almost no differentiation between the aspects at 50 ms post-stimulus instance as well as at 104 ms post-stimulus. This processing overlap was attenuated at 200 ms meaning that distinct brain regions have become involved in the processing of distinct aspects of information.

I also defined and calculated the information-selectivity index across time (Fig. 7B). The information-selectivity index was calculated on each information-selectivity matrix as the average of within-aspect correlation coefficients (i.e. the average of all correlation values obtained within categorization, lighting, pose, size and position processing) minus the average of between-aspect correlation coefficients (e.g. the average of correlation values across categorization and lighting processing, etc.). The time instances of the information-selectivity matrices, shown in Fig. 7A, are highlighted by red arrows in Fig. 7B. The information-selectivity curve revealed its first and second significant (p < 0.05, Wilcoxon’s signed-rank test) declines respectively in the time spans from 43 to 61 ms and from 101 to 113 ms post-stimulus. These significant declines totally matched the occipital co-processing of category and variation information shown in Figs 5 and 6. Interestingly, these time spans also highly concurred with the first stage of visual processing (Fig. 4A, right) in which information regarding categories and variations were processed in an in-phase pattern which together support the spatiotemporal co-processing of category and variation information in early visual cortices (Fig. 5). During the second stage of visual processing (from 170 to 450 ms post-stimulus), in which category and variation information were processed in an anti-phase pattern (Fig. 4A, right), distinct brain mechanisms were involved in the processing of distinct aspects of information (Figs 5 and 6). Together, these quantitative results suggested that, in the earliest stages of sensory processing, information about categories and variations were processed by similar neural mechanism and in later time windows, the information-selective brain areas undertook their intrinsic tasks.

### Transaction of visual information between peri-occipital and peri-frontal areas

**Figure 8:**
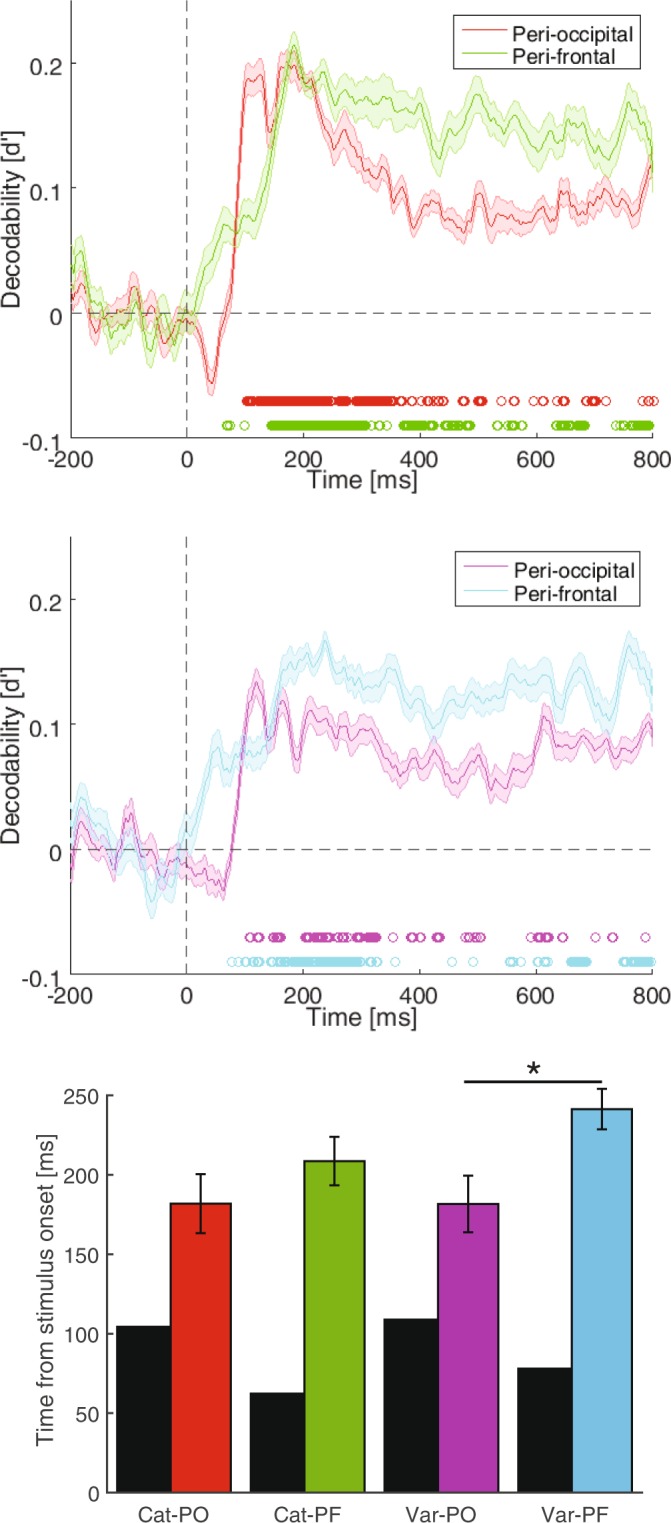
Time-resolved decodability of categories (top), variations (middle) and their temporal statistics (bottom) in the peri-occipital and peri-frontal areas. The circles indicate the time points at which the color-matched decodability curves were significantly above the decodability values averaged in the last 200 ms pre-stimulus window (i.e. p < 0.05, FDR corrected Wilcoxon’s signed-rank test). (**C**) Latency (black) and peak (colored) time points of decoding in corresponding category and variation decodability curves. Shaded areas and error bars indicate the SEM across subjects. Stars show significant (p < 0.05, Wilcoxon’s signed-rank test) difference between peak time bars. ‘Cat’ and ‘Var’ respectively stand for categories and variations while ‘PO’ and ‘PF’ respectively represent peri-occipital and peri-frontal.

Although the above analyses provided new insights into the distinct stages of category and variation processing in the brain, they remained silent on the possible flows of information between brain areas. Recent studies have suggested that specific properties of objects (e.g. low-frequency components of the object image) were processed by mechanisms of prefrontal cortex in parallel to the ventral visual stream whose results are transferred from lower (i.e. V1) to higher visual areas such as IT^14,19^. These studies and other theoretical and experimental investigations, which suggested that some variations may need top-down prefrontal-to-occipital feedback signals for accurate recognition^20,29,60^, provided motivation to evaluate the possible transfer of category and variation information between the peri-frontal and peri-occipital areas in object processing. For that purpose, the information processing units of peri-frontal and peri-occipital areas were separated by electrodes (Figs 8 and S1B). Results showed an earlier rise to significance (p < 0.05) on peri-frontal electrodes about both categories and variations (respectively at 68 and 78 ms) compared to peri-occipital electrodes (respectively at 103 and 108 ms) (Fig. 8, top and middle). However, information about categories and variations in peri-occipital areas peaked earlier than those in peri-frontal areas (Fig. 8, bottom). These results suggest that, in contrast to what might be expected regarding the dominant role of posterior brain areas in the processing of category and variation information, the same number of electrodes on peri-frontal areas can provide even higher amounts of information compared to peri-occipital areas, especially at later stages of processing. The earlier rise of information at frontal brain areas can be explained by the magnocellular projections from the eyes to the frontal brain areas which provide a faster parallel pathway to those from the eyes to the occipital lobe. This is explained in more details below.

What remains unknown is whether there was any interaction of information between frontal and occipital brain areas which is the subject of following analyses. To that end, I evaluated the spatiotemporal dynamics of information transfer between peri-occipital (including occipital and parietal electrodes of O1, O2, OZ, POZ, P3, P4, P7, P8 and PZ) and peri-frontal (F3, F4, F7, F8, FZ, AFZ, FP1, FP2 and FPZ) areas using a simplified version of Granger causality as suggested previously^19^. For that purpose, first I calculated similarity matrices at every time point using the 9-dimentional representational space (i.e. nine electrodes) for object categories and variation conditions. Then, using partial correlations between representations at time *t* and the average of representations in the span from *t* − 130 to *t* − 80 ms, I investigated the transfer of information between peri-frontal and peri-occipital brain areas (see Methods and Goddard *et al*.^19^ for more information). I called the information directions from peri-occipital to peri-frontal “feed-forward” and from peri-frontal to peri-occipital as “feedback” anatomically and not based on the classical feed-forward and feedback flows of visual information which is dominant in the literature. In other words, rather than the role of peri-frontal areas in higher level cognitive processes such as attention, decision making, etc.^34,35^, I am investigating the role of specific compartments within those areas such as orbitofrontal cortex in providing a parallel processing pathway to sensory object processing areas such as occipitotemporal cortices^13,14,19^. This is discussed in more details in Discussions.

**Figure 9:**
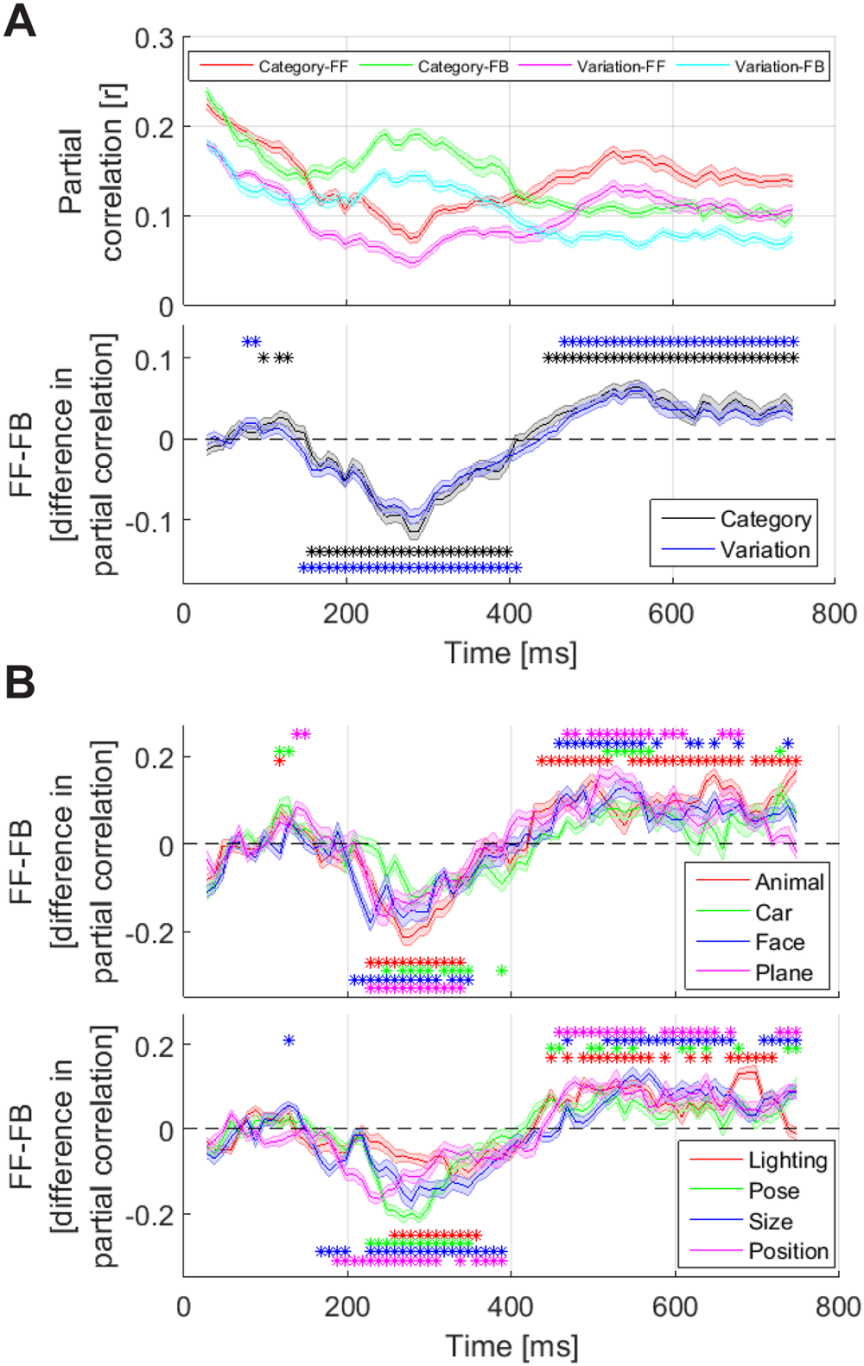
Time-resolved flows of category and variation information in the brain. (**A**) Top, partial correlation of representations between peri-occipital and peri-frontal areas. FF and FB refer to the feed-forward (correlation between time *t* representations in peri-occipital and representations during time *t* − 130 to *t* − 80 ms in peri-frontal areas) and feedback information flows, respectively. (**A**) Bottom, the difference between FF and FB flows of information for categories (black) and variations (blue). Stars indicate the time points at which the flows were significantly higher (p < 0.05, random permutation test) than correlations obtained from a null distribution. (**B**) The same as (**A**, Bottom) but for each category (top) and each variation (bottom) with their corresponding significant time points indicated with stars. Shaded areas indicate the SEM across subjects.

Partial correlations between peri-frontal and peri-occipital information showed higher values for category than for variations (compare the red and green curves with cyan and magenta curves on the top panel of Fig. 9A). In order to measure the dominance of information flow on the scalp, I calculated the difference between feed-forward and feedback information (i.e. between partial correlations, Fig. 9A, bottom) and evaluated the significance of the calculated differences against 1000 randomly generated partial correlation values (see Methods). The difference information curves showed highly dynamical patterns switching from feed-forward to feedback at around 150 ms and reversing to feed-forward at around 420 ms for both categories and variations. Results showed that variation and category information led to significant feed-forward flows respectively at 77 and 97 ms and remained significant respectively until 88 and 128 ms post-stimulus. Then, the variation and category information turned into significant feedback flows at 147 and 158, remained significant respectively until 408 and 397 ms, turned into feed-forward flows again respectively at 469 and 448 ms and remained significant until the end of analysis time. Therefore, information regarding categories and variations moved dominantly from peri-occipital areas towards peri-frontal areas in the window from the stimulus onset to 130 ms (first stage) post-stimulus, then back to peri-occipital areas from around 150 ms to 400 ms (second stage) and then again forth to peri-frontal areas from around 450 ms (third stage). The observed stages of category and variation processing confirmed many of the results reported above, as follows. The first stage, which supported feed-forward flows of information from peri-occipital to peri-frontal areas (Fig. 9A, bottom) co-aligned with the in-phase (Fig. 4A) entangled (Fig. 7B) processing of category and variation information. The second stage, which concurred with feedback flows of information from peri- frontal to peri-occipital areas (Fig. 9A, bottom), covered the anti-phase (Fig. 4A) information- selective (Fig. 7B) stage of category and variation processing. The third stage, which again revealed feed-forward flow of information from peri-occipital to peri-frontal areas (Fig. 9A, bottom), covered the in-phase (Fig. 4A) information-selective processing windows explained above (Fig. 7B). The observed temporal dynamics of information flows were also highly consistent with a seminal study which reported feed-forward flow of object information from subcortical/occipital (e.g. V1) areas to orbitofrontal cortex at around 80 ms (i.e. the start time of the first stage of processing in the current study) followed by the feedback of category information starting at around 130 ms (i.e. the start time of the second stage of processing of the current study) post-stimulus onset^14^.

To be able to compare the dynamics of information flow across categories and variations, I provided category and variation information flows resolved into their constituent conditions (Fig. 9B). No significant differences were observed between categories (Fig. 9B, top). However, as previously suggested^8^, pose seems to have employed a higher level of feedback compared to other variations (Fig. 9B, bottom). More importantly, while category information has provided a higher number of significant feed-forward time points during the first stage, variations have employed a wider span of significant information feedback.

### Comparing the brain’s dynamical behavior with a computational model

**Figure 10:**
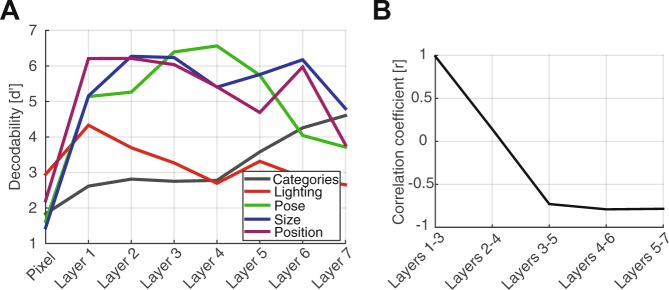
Correlation of the model with brain data. (**A**) Decodability indices obtained from pixel-space images as well as their representations at the output of every layer of the model for categories (black curve) and variations (color curves). (**B**) Correlation values between the average of four variation indices and the category index at three consecutive layers of the model (i.e. three data values were used in the calculation of correlations) which showed significantly (p < 0.05, Pearson linear correlation) positive and negative values respectively in the first-half and second-half of the model layers.

A set of computational models of human visual processing have been proposed recently which were able to provide accurate prediction of object representations at final layers of the ventral visual stream^47,48,51^ (V4 and IT). One of these models, HMO, which had a hierarchical feed-forward structure, has recently suggested that information regarding both categories and variations were enhanced as object representations passed through layers of the model^61^, supporting that a unified structure can process category and variation information by entangled mechanisms. However, as here I supported the existence of parallel pathways for visual object representations, it was interesting to know how one of the most brain-plausible versions of such hierarchical models, known as ‘AlexNet’^51^, would process category and variation information. To that end, I fed the model with the same image set as was used in the EEG experiment and measured the decodability indices across categories and variation conditions at the output of every model layer (Fig. 10A). Information about categories and variations increased as object images passed the first layer of the model. Except for lighting which showed a monotonically decreasing information curve, other variations generally experienced information enhancement by going from the first to the fourth layer of the model. After the fourth layer, the variation decodability indices decreased while the category information kept increasing until the last layer of the model. Therefore, two distinct stages of information processing seemed to be at work in the model: one from the first to the fourth model layer and one from the fourth to the last layer. In order to evaluate the relative phase of category and variation decodability in the model, I calculated the correlation of decodability patterns in every three consecutive model layers between category and the average of four variation decodability curves (Fig. 10B), which showed an in-phase followed by an anti-phase processing pattern. These two stages seem to repeat the first and second stages of visual processing obtained from EEG signals (Fig. 9). The third stage of processing, however, was absent from the computational model, which seems to be a result of the model lacking the decision-related mechanisms present in the brain, as it was most probably the destination of information flow during the third stage of visual processing (i.e. PFC). Therefore, the hierarchically-organized feed-forward model of visual processing which was used here seemed to be a brain-plausible model which implemented the existing parallel visual pathways (i.e. one going from V1 to orbitofrontal cortex and back to IT and the other directly from V1 to IT) that process visual information prior to the convergence of information at IT cortex. The parallel processing structures of the model were probably implemented by different convolutional spatial filters each of which extracted and processed different sub-band frequencies of the visual input which had been inspired by the brain mechanisms for filtering different spatial frequencies^14,19^.

I also evaluated the spatiotemporal correlation between the category information in the EEG signals, whose results reflected the hierarchical structure of computational models (Supplementary Fig. S4). Results of this analysis confirmed the existence of a hierarchical structure for category processing in the brain, as previously observed for the same model^62^. In fact, the results showed that a layer-wise structure could have underlay the observed EEG decodability indices, but does not rule out the possibility of parallel visual processing being at work in peri-frontal and peri-occipital areas. Next, using the representational vectors which were obtained from the last layer of the computational model on an extended version of the current image set^8^, the representational dissimilarity matrices also showed the distinctiveness of face category exemplars from the other categories validating the results explained for Fig. 2A, right (Supplementary Fig. S5A, left). The variation representational dissimilarity matrix also showed the decodeability/distinctiveness of different variation levels (size and pose conditions, Supplementary Fig. S5A, right). Finally, using the same extended image set^8^ and the representations obtained from the last model layer, I also showed that variations in pose could drastically entangle object representations, whereas lighting had little impact on object representations (Supplementary Figs S2C and S5B). This was also reflected in the behavioral object recognition performance of my recent work (Supplementary Fig. S5B, light-colored images)^8^. Therefore, although they may divide the visual object processing problem into several sub-problems (e.g. by using sets of convolutional filters with different shape- and frequency-based sensitivities), which may not necessarily follow those implemented by the brain (e.g. dividing the problem into low- and high-frequency components in frontal and occipital brain areas)^63^, the recently developed computational models of human vision can be proper candidates to access primate’s high-level visual representations.

## Discussions

This investigation provides a broad-based survey of the spatiotemporal dynamics of category and variation processing in the human brain as well as their interactions. Findings of this study provided several insights. First, the visual processing of category and variation information was shown, for the first time, to be divided into three stages (Figs 7–9): stage one, which covered the time window before 130 ms post-stimulus during which information about categories and variations were processed by (temporally and spatially) entangled mechanisms mainly concentrated in primary visual cortex and were simultaneously (significantly during 80 to 130 ms window) sent to the frontal brain areas^13,14^; stage two, which covered the time window from 150 to 400 ms post-stimulus during which category and variation information, being processed by partially anti-phase distinct mechanisms, were sent back to peri-occipital areas; and stage three, which started at around 450 ms with category and variation information sent, in a temporally in-phase, to frontal areas possibly for final category-related decisions. Second, this study provided experimental support that information regarding categories as well as variations were processed also in the peri-frontal areas of the brain (Fig. 5). This added evidence to the previously suggested role of frontal brain areas in the processing of low-frequency object information^14,19^. Third, as subjects’ task was unrelated to object recognition (as a result of passive paradigm), it could be concluded that the observed spatiotemporal dynamics of category processing have been mainly driven by the stimulus presentation rather than being task-driven. Finally, the results showed that a feed-forward convolutional neural network model could predict the first two above-suggested stages of visual processing implying that rather than feedback, the second stage of visual processing could support the existence of a processing pathway operating in parallel to the known processing stages implemented by the ventral visual stream (from V1 to IT).

Although a few recent studies have observed the information regarding the processing of variations in the human brain^19,23,64^, current study is the first to investigate the whole-brain spatiotemporal dynamics of affine (i.e. size and position) and non-affine (i.e. lighting and pose) variation processing using a systematically designed image set. These results have extended previous findings which concentrated on specific variations (e.g. position^28^), evaluated limited areas of the brain (e.g. V4 and IT^23^), used low temporal resolution recording methods (e.g. fMRI^64,65^) or overlooked possible flows of variation information in the brain^19^. Through different analyses (Figs 4–9), this study provided support that significant processing of information about categories and variations was initiated (before 130 ms post-stimulus) in the primary visual cortex and the information was simultaneously sent to peri-frontal areas. This entangled processing of category and variation information was also reported by previous studies which suggested the processing of category and variations in alternating layers of the ventral visual stream^23,64^. In the later window (from 150 to 400 ms), which overlapped with the peri-frontal to peri-occipital window of information flow (Fig. 9A) previously observed for impoverished objects^66^ and attended object detection^67^, variation and category information showed spans of in-phase and anti-phase processing patterns (Fig. 4A, right). The relationship between the category and variation phase patterns (Fig. 4A, right) and the direction of information flow (Fig. 9A, bottom) suggested distinct mechanisms for transferring information from occipital to frontal areas and back to peri-occipital areas. Previous studies have suggested that the information from early visual cortex travels through fast dorsal magnocellular pathway to reach OFC^14,15^. The frontal-to-occipital information, however, may use slower pathways which start from frontal cortex and end at occipitotemporal and temporal areas^17,18,38,67–69^. The final window (starting from 450 ms), showed simultaneous processing and transferring of category and variation information from occipital to frontal areas. During this final window, the representational information was probably transferred to peri-frontal areas for final cognitive processes such as decision-making and response preparation^70,71^.

It should be noted that the aim of current study was to compare the spatiotemporal dynamics of variation against category information processing in the brain, and not to address the invariance problem which has been previously addressed by several studies^25,54^. In Isik *et al*.^54^, the dynamics of object processing under variations of size and position were evaluated which was extended by a later study to the variations of pose and lighting^8^. On the other hand, in the current study as well as several recent studies^23,72^, the spatiotemporal dynamics of individual variations were evaluated separately from the information of categories.

Although several studies have recently proposed the contribution of peri-frontal areas (LOC) to the encoding of low-frequency object information^14,19^, the current study seems to be the first to show that information regarding variations was also processed by peri-frontal areas. It did not only show that peri-frontal areas contributed to variation processing, but also revealed that this area sent variation information to occipital areas. This implies that, in contrast to the consensus that the ventral and dorsal visual streams dominate the processing of category and variation information by feed-forward mechanisms^73,74^, the representations in peri-frontal cortex can provide even larger amounts of information compared to those areas in large portions of the processing window (Fig. 8, compare the time windows after 200 ms and a related study which showed the same temporal dynamics for feedback signals^66^). Results also showed an earlier (from 0 to 50 ms) rise of information curves in peri-frontal than in peri-occipital areas (Figs 4B,C and 8) which can be explained by a previously suggested “framing model”^18,75^. In this model, a frame of the object, which is a vague low-frequency representation or a gist of it, is constructed in peri-frontal brain areas (through subcortical pathways from the eyes to the orbitofrontal cortex), enhanced by the peri-occipital to peri-frontal flows through magnocellular pathways and is fed back during the second stage of processing to enhance the details of object representations for accurate recognition^67^.

As I did not separate the low- and high-frequency components of the objects, it could not be determined which object features were processed by the peri-frontal areas. For variations, however, an advantage was observed in the processing of variations of lighting and pose (compared to variations in size and position) in the peri-frontal areas compared to peri-occipital areas throughout the processing time (Fig. S3B). This is on par with previous suggestions that frontal areas may play role in the compensation of non-affine variations^8,29^.

An interesting implication of the current results was that the observed role of peri-frontal brain areas in object and variation processing seem to be an integral part of visual processing rather than being activated by top-down cognitive processes which generally become involved during active object recognition. In fact, as the current paradigm was irrelevant to object recognition, the contribution of frontal brain areas could not have been mainly driven by the subject’s task. This aspect of the results, while supporting previous findings on the role of peri-frontal cortex in active object recognition^13,14,19,66,67^, has extended those results to the general case of object processing as opposed to object recognition which can be highly affected by the task, context, etc.^63,76–78^. This result is on par with a recent study which has suggested that the activation of orbitofrontal cortex is independent of the task, the characteristics of the visual input and explicit recognition of the stimuli^75^.

The two-stage processing of variations observed in the computational model (Fig. 10, stage one: layers 1–4 and stage two: layers 5–7) can be explained by the dimensionality of the model’s representational space at different model layers. In other words, a very high correlation (r = 0.7117, p = 0.0728, n = 7; Pearson linear correlation) was observed between the size of the representational space (i.e. which were 69987, 43264, 64896, 64896, 9216, 4096 and 4096 respectively for layers 1 to 7) and the decodability indices across model layers (Fig. 10A). The correlation was much less for lighting alone (r = 0.4876, p = 0.267) which was probably a result of lighting (as a low-level image feature) being mainly compensated for by the first layer of the model. Moreover, the category decodability curve and the model’s representational dimensions showed an anti-correlated pattern (r = −0.9231, p = 0.003). It can be concluded that, as suggested by the idea of population coding of variations^79^, large neural populations may be proper candidates to compensate for object variations, while for the encoding of categories, the brain may exploit its sparse inter-layer connections (as implemented in the two final fully-connected layers of the computational model^51^).

While some recent studies have reported correlated processing stages between the human brain and those computational models (as in Fig. S4)^23,62^ especially at higher visual areas^47,48^, they have overlooked possible parallel processing mechanisms that could have contributed to those correlated patterns at final layers. In other words, computational models and the brain might have implemented different sets of strategies to reach the same abstract representations of objects found in their final processing stages as previously proposed^63^. Therefore, even a layer-wise correlation does not rule out the possible existence of parallel, interactive mechanisms for object processing in the brain.

One advantage of this study to a most relevant study, which supported the role of peri-frontal areas in object recognition^19^, is that it has used a rapid presentation paradigm in which objects were presented only for 50 ms. This was important since longer presentation times could have caused the dominance of peri-occipital to peri-frontal information (referred to as feed-forward) flow compared to the peri-frontal to peri-occipital (feedback) flow, leading to the underestimation of feedback influence. As argued by the authors^19^, the same reason may explain the dominance of feed-forward as well as the lag of feedback information flows in that study. However, these results suggested the need for a systematic study to investigate the impact of presentation time on the amplitude and the temporal dynamics of object information flow in the brain.

While the results of current study have provided new insights into the spatiotemporal dynamics of category and variation processing in the human brain, they raised several questions. Does the task affect the processing of variation information differently from category information? It has been recently shown that the task can significantly modulate category representations in high-level vision-related areas^76^ which can result in the modulation of processing strategies^63^. It remains to be studied for variations as well. Second, is the variation information, observed here, also observed when objects are presented on complex backgrounds (i.e. clutter)? Third, what exactly are the regions which process category and variation information in peri-frontal areas? Although there are suggestions on the role of orbitofrontal cortex in that regard, more accurate recording methods (e.g. combined EEG and fMRI) can tell if information about categories and variations are processed by the same or different brain regions. Regardless of how these questions are addressed, the current study provides new insights into the role of peri-frontal brain areas in category and variation processing.

## Electronic supplementary material

SUPPLEMENTARY INFO

## Data Availability

The datasets generated during and/or analyzed during the current study are available from the corresponding author on reasonable request.
