# Supplementary material for: Three-stage processing of category and variation information by entangled interactive mechanisms of peri-occipital and peri-frontal cortices
Source: Sci Rep. 2018 Aug 15;8:12213. doi: 10.1038/s41598-018-30601-8 (PMC6093927; doi:10.1038/s41598-018-30601-8)
Supplement: Supplementary file 1 — SUPPLEMENTARY INFO [file 41598_2018_30601_MOESM1_ESM.pdf]

Supplementary Information for:

Three-stage processing of category and variation information by entangled interactive mechanisms of peri-occipital and peri-frontal cortices

Hamid Karimi-Rouzbahani

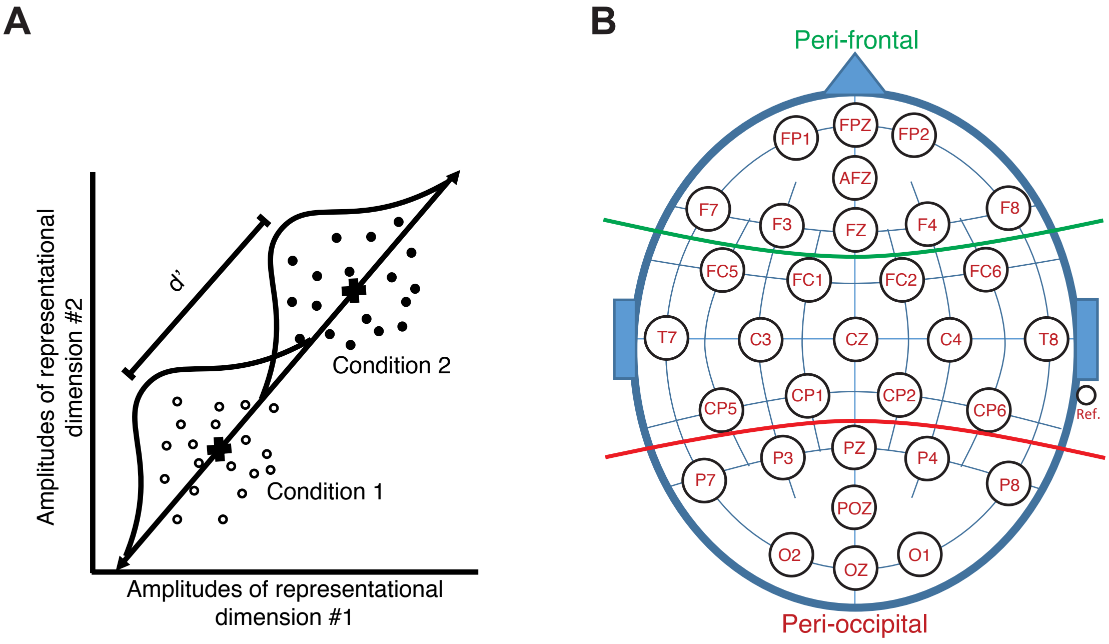

Fig. S1. The representational space for decodability analysis (A) and EEG electrode locations (B). Peri-occipital and peri-frontal areas are also indicated with red and green curves in (B).

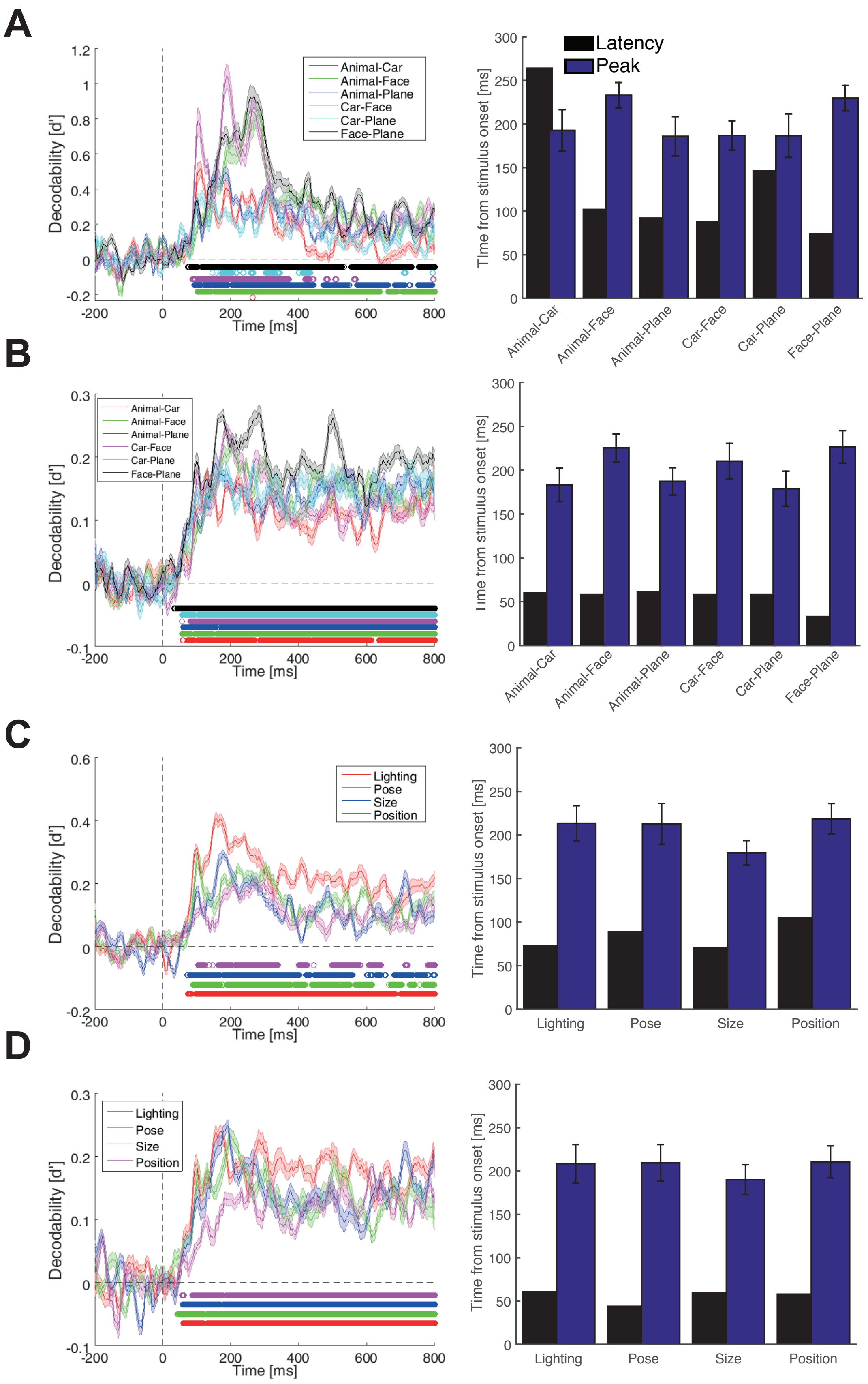

Fig. S2. Across-time decodability of category pairs and categories under variations. Decodability curves in (A) and (B) show the across-category results respectively in the pooled- and per-condition cases. Decodability curves in (C) and (D) show the category representational analysis results under each of the variations respectively in the pooled- and per-condition cases. The bar plots provide latency (blue) and peak times (black) of the left side decodability curves.

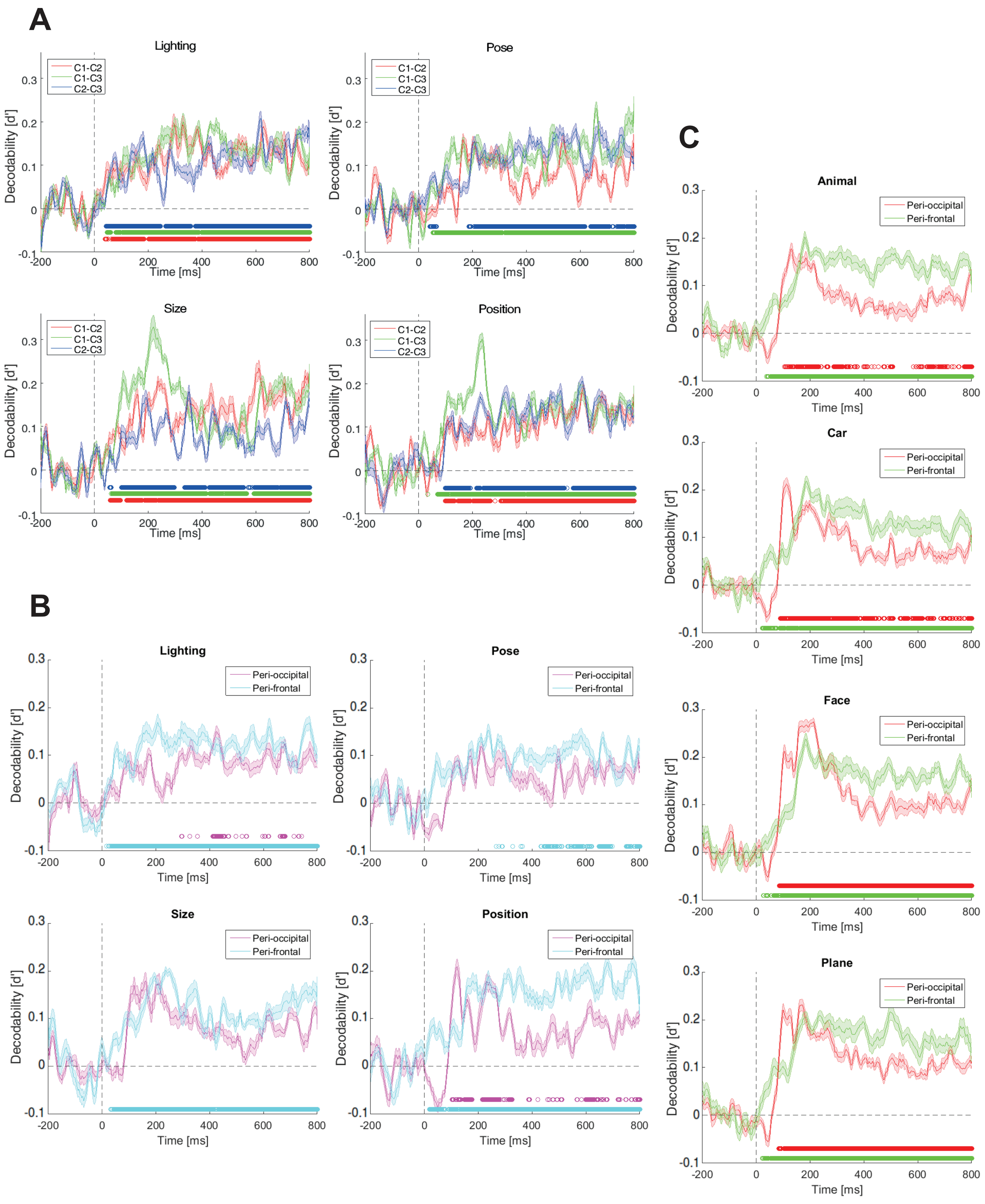

Fig. S3. Across-time analysis of variation and category decodability. (A) Decodability of variation-conditions. (B) and (C) respectively provide decodability indices of variations and categories in peri-frontal and peri-occipital brain areas. The vertical and horizontal dashed lines indicate respectively the stimulus onset time and the unity decodability value. The circles indicate the time points at which the color-matched decodability curve was significantly above the decodability values averaged in the last 200 ms pre-stimulus (i.e. p < 0.05, evaluated using Wilcoxon's signed-rank test). Shaded areas and error bars indicate the SEM across subjects.

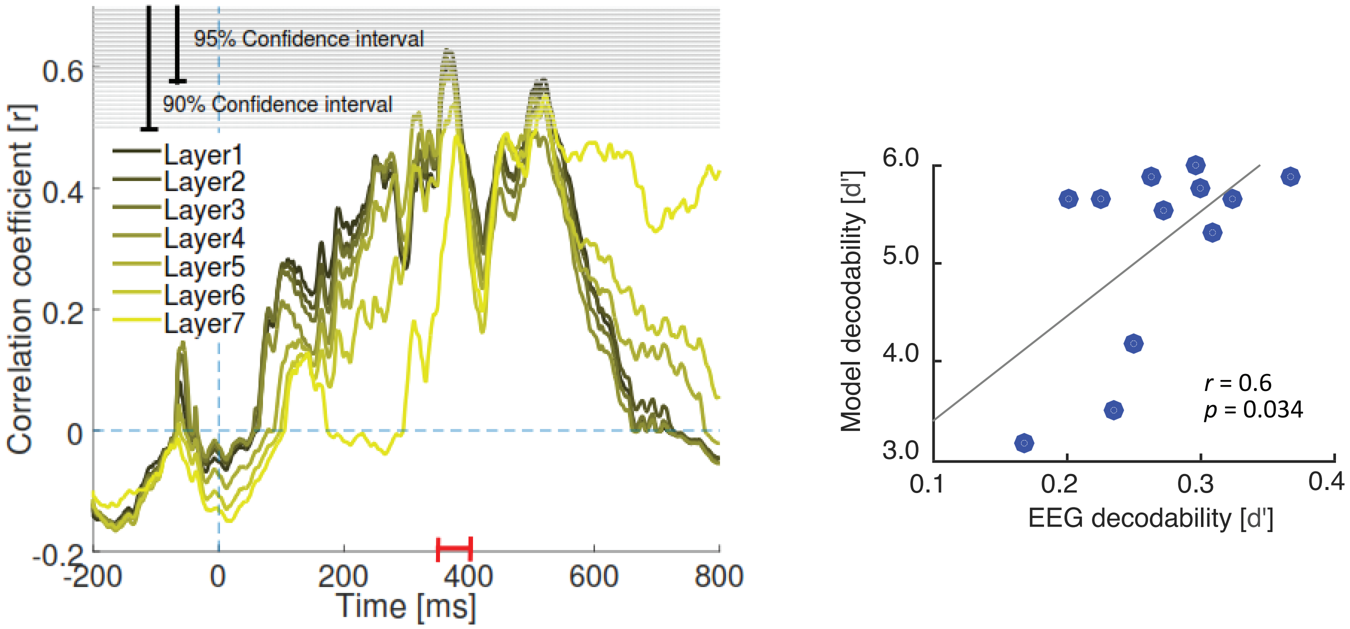

Fig. S4. Across-time correlation between category decodability indices obtained from model layers and whole-brain EEG signals. Correlation coefficients (Pearson linear correlation) were calculated between 50 ms moving average windows on the 12 decodability indices (i.e. the decodability index fobtained rom each variation condition) of the brain and the 12 decoding indices obtained from different model layers on each variation condition. Significance of correlations is indicated by shaded areas for p < 0.05 (confidence interval = 95%) and p < 0.1 (confidence interval = 90%). The scatter plot on the right shows the distribution of decodability indices obtained from model layers (averaged across layers) and whole-brain EEG signals in the window from 350 to 400 ms post-stimulus where they model and the brain showed the highest correlation values (indicated by the red bracket in the left plot). The solid line shows the best first-order linear fit to the data points. As these results show, positive correlations started to appear between different model layers and the EEG decodability indices at around 60 ms after the onset of the stimuli. The correlation coefficients reached their first significant peak (p < 0.05 for layers 1 to 5 and p < 0.1 for layer 6) at around 360 ms, experienced their second significant peak (p < 0.05 for layer 1, p < 0.1 for layers 2,3,5,6 and 7, Wilcoxon's signed rank test) at 515 ms and started to descend at 600 ms post-stimulus. Interestingly, the initial model layers (layers 1 and 2) started to become correlated to the EEG sooner than the middle layers (i.e. layers 4 ,3 and 5) and in turn the middle layers before the final layers (i.e. layers 6 and 7); the first time point at which the correlations surpassed the value of 0.1 for layers 1 to 7 were respectively 114 ,100 ,72 ,70 ,69 ,70 and 120 ms. Although all layers experienced the same two peaks, the difference is clear between the rising pattern of the initial layers and the final layers. Moreover, the initial layers showed a higher value in the first peak than in the second peak while it was the opposite for the final layers with higher values in their second peaks. These results provide evidence for a hierarchical feed-forward structure to be underlying the brain representations of objects in the current study.

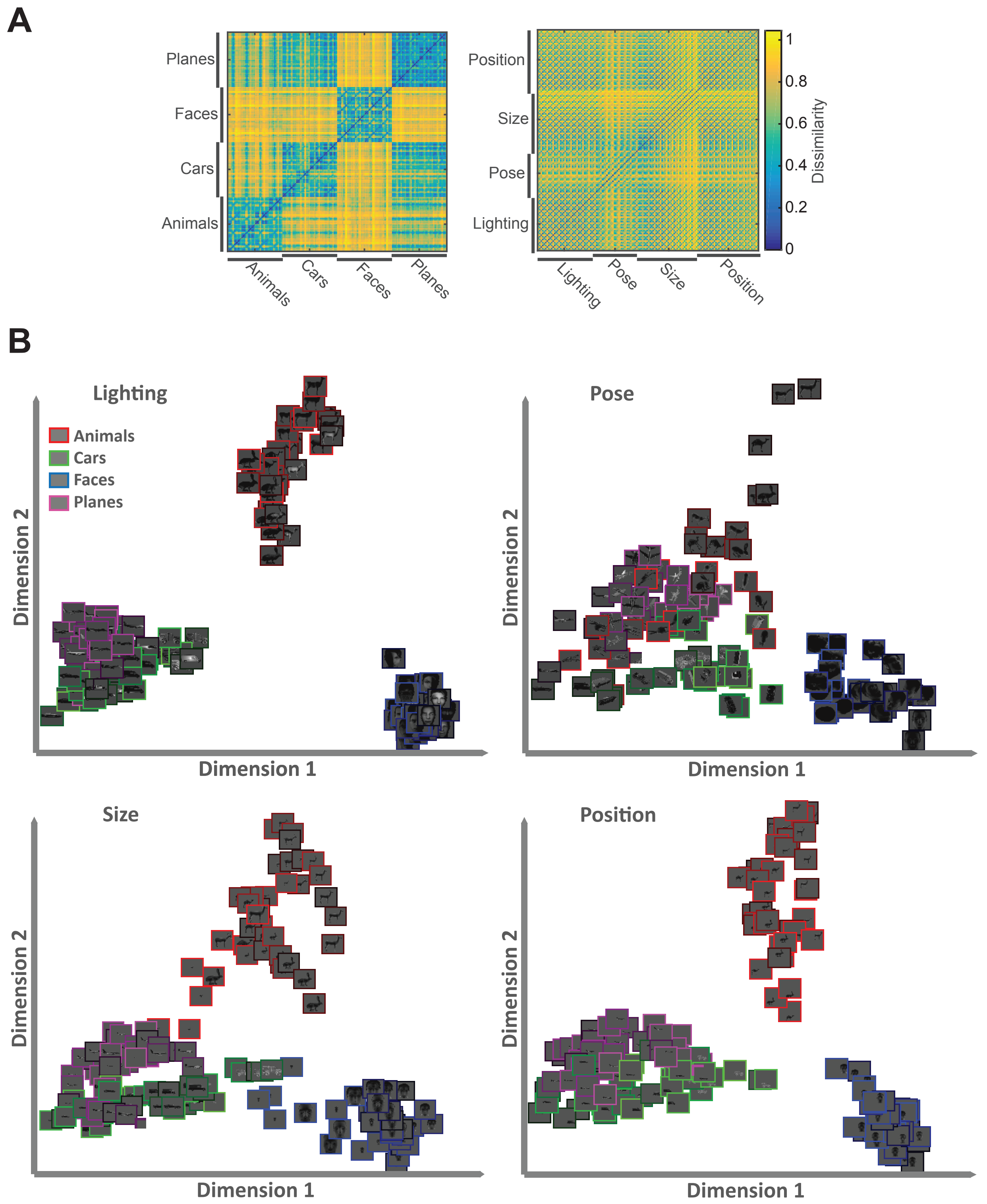

Fig. S5. Model representations of the whole image set. (A) Representational dissimilarity matrices show across-stimulus correlation between representations obtained from last model layer, sorted by their categories (left) and variation conditions (right). Warmer colors indicate higher correlations. (B) 2D multi-dimensional scaling (MDS) plots show how objects scattered relative to one another under the influence of each variation on the model's output. Animal, car, face and plane exemplars are clustered at the top, bottom left above, bottom right and bottom left under, respectively in the plots. The level of brightness for the edge colors in animal, car, face and plane categories which are respectively red, green, blue and magenta, was determined based on behavioral categorization accuracy reported in my previous paper (Karimi-Rouzbahani et al., 2017b). For instance, the edge color of a human face exemplar in the 2nd pose condition is in brighter blue than the same human face on the 5th pose condition if the human subjects categorized faces more accurately in the 2nd pose condition compared to the 5th. The representations are from the 7th model layer. The color-coding scheme allows you to investigate the relationship between the position of representations and behavioral results. Category clusters were positioned more separated under the variations of lighting and position than under pose and size variations. In the lighting conditions, no definite conclusions can be made for different exemplars as a result of descent untangling. For pose variation, however, exemplars with brightly-colored edges are from conditions in which the objects underwent a high level of in-depth orientation (e.g. objects are in profile, top or back views).

In other words, as the objects underwent in-depth orientations, their representations moved out of their category clusters, approached other category clusters and entangled with them which in turn made the categorization difficult. For size, the majority of bright-edged exemplars are from small-sized objects which take positions near irrelevant clusters, while large-sized exemplars are far from other clusters. The same is true for position, in which images with more eccentric objects in the central representational space approach other clusters which made it difficult for categorization (as shown with bright edges), while the images with more central objects remain at the furthest positions relative to other categories. Therefore, I can see a clear effect of variations on the representational space along with several well-known properties of category encoding in the brain which are mentioned below. Face exemplars positioned more closely compared to other categories which can be another proof for a specialized selectivity to faces higher visual areas (Freiwald and Tsao, 2010). Moreover, car and plane categories kept closer distance to one another compared to other categories which can be explained by both their similarity from perceptual and semantic respects. Together it can be concluded that category and variation information shape the brain's representational space in a cooperative manner. The 3D models used to generate the above object images were available under a personal and commercial license (http://www.cadnav.com/help/copyright.html) and were freely downloaded from (http://www.cadnav.com).
